# Investigating the causal relationship between ankylosing spondylitis and osteoporosis in the European population: a bidirectional Mendelian randomization study

**DOI:** 10.3389/fimmu.2023.1163258

**Published:** 2023-06-08

**Authors:** Jian Mei, Hongxin Hu, Haiqi Ding, Ying Huang, Wenming Zhang, Xiaoqing Chen, Xinyu Fang

**Affiliations:** ^1^ Department of Orthopaedic Surgery, Fujian Provincial Institute of Orthopedics, The First Affiliated Hospital of Fujian Medical University, Fuzhou, China; ^2^ Department of Orthopaedics, Affiliated Hospital of Putian University, Putian, China; ^3^ Department of Orthopedic Surgery, Experimental Orthopedics, Centre for Medical Biotechnology (ZMB), University of Regensburg, Regensburg, Germany; ^4^ Department of Orthopaedic Surgery, National Regional Medical Center, Binhai Campus of the First Affiliated Hospital, Fujian Medical University, Fuzhou, China; ^5^ Department of Orthopedic Surgery, Quanzhou First Affiliated Hospital of Fujian Medical University, Quanzhou, China

**Keywords:** Mendelian randomization, ankylosing spondylitis, osteoporosis, bone mineral density, single nucleotide polymorphisms, GWAS

## Abstract

**Background:**

Ankylosing Spondylitis (AS) is an inflammatory condition affecting the spine, which may lead to complications such as osteoporosis (OP). Many observational studies have demonstrated a close relationship with strong evidence between OP and AS. The combination of AS and OP is already an indisputable fact, but the exact mechanism of AS complicated with OP is unclear. To better prevent and treat OP in patients with AS, it is necessary to understand the specific mechanism of OP in these patients. In addition, there is a study showing that OP is a risk factor for AS, but the causal relationship between them is not yet clear. Therefore, we conducted a bidirectional Mendelian randomization (MR) analysis to determine whether there is a direct causal effect between AS and OP and to investigate the co-inherited genetic information between the two.

**Methods:**

Bone mineral density (BMD) was used as a phenotype for OP. The AS dataset was taken from the IGAS consortium and included people of European ancestry (9,069 cases and 13,578 controls). BMD datasets were obtained from the GEFOS consortium, a large GWAS meta-analysis study, and the UK Biobank and were categorized based on site (total body (TB): 56,284 cases; lumbar spine (LS): 28,498 cases; femoral neck (FN): 32,735 cases; forearm (FA): 8,143 cases; and heel: 265,627 cases) and age (0-15: 11,807 cases; 15-30: 4,180 cases; 30-45: 10,062 cases; 45-60: 18,062 cases; and over 60: 22,504 cases).To obtain the casual estimates, the inverse variant weighted (IVW) method was mainly used due to its good statistical power and robustness. The presence of heterogeneity was evaluated using Cochran’s Q test. Pleiotropy was assessed utilizing MR-Egger regression and MR-pleiotropy residual sum and outlier (MR-PRESSO).

**Results:**

Generally, there were no significant causal associations between genetically predicted AS and decreased BMD levels. The results of MR-Egger regression, Weighted Median, and Weighted Mode methods were consistent with those of the IVW method. However, there was a sign of a connection between genetically elevated BMD levels and a decreased risk of AS (Heel-BMD: OR = 0.879, 95% CI: 0.795-0.971, *P* = 0.012; Total-BMD: OR = 0.948, 95% CI: 0.907-0.990, *P* = 0.017; LS-BMD: OR = 0.919, 95% CI: 0.861-0.980, *P* = 0.010). The results were confirmed to be reliable by sensitivity analysis.

**Conclusion:**

This MR study found that the causal association between genetic liability to AS and the risk of OP or lower BMD in the European population was not evident, which highlights the second effect (e.g., mechanical reasons such as limited movement) of AS on OP. However, genetically predicted decreased BMD/OP is a risk factor for AS with a causal relationship, implying that patients with OP should be aware of the potential risk of developing AS. Moreover, OP and AS share similar pathogenesis and pathways.

## Introduction

Ankylosing spondylitis (AS) is an autoimmune disease characterized by chronic inflammation and new bone formation involving the central axis bone and sacroiliac joints, along with peripheral joint involvement and extra-articular involvement, which can ultimately result in inflammatory low back pain and spinal limitations. Approximately one-third of AS patients can be incapacitated (1, 2). AS can affect individuals of any age, but it is most commonly diagnosed in individuals between the ages of 20 and 30 years, with a male-to-female ratio of approximately 2:1 (1). However, research suggests that the proportion of patients with AS who are over 60 years old and the proportion of women with the condition are increasing (2, 3). The prevalence of AS varies by region and ethnicity and is estimated to range from 0.1% to 1.4% (4). For example, the average prevalence of AS in the European population is 23.8 cases per 10,000 individuals (4). Furthermore, a retrospective cohort study based on the Korean population found that from 2010 to 2015, the annual linear increase in AS incidence was 7.7%, rising from 31.62 to 52.30 cases per 100,000 individuals (5). The study also found that the incidence of AS in male patients peaked in the 20-29 years age group, while the incidence in female patients peaked in the 70-89 years age group (5).

Osteoporosis (OP) is a chronic metabolic bone disease that is most commonly diagnosed through dual-energy X-ray absorptiometry (DXA) and is characterized by decreased bone mineral density (BMD) in clinical settings (6). OP is prevalent globally, affecting approximately 200 million individuals, with the incidence increasing with age and being more common among women (7). Genetic factors, lifestyle, and medical history are also important factors that influence the incidence of OP (8). Early diagnosis and treatment of OP can help reduce the risk of fractures and improve quality of life.

Both AS and OP have strong genetic components. AS is strongly associated with the HLA-B27 genotype, which is present in up to 90% of patients with AS. In addition, genes related to the immune system and inflammation response, such as IL-23R, ERAP1, and TNFRSF1A, can also impact the occurrence, progression, and severity of AS (9–12). OP is a multifactorial disease involving multiple genes and diverse modes of inheritance, primarily associated with BMD. Many genes, including low-density lipoprotein receptor-related protein 5 (LRP5), Sclerostin (SOST), Wnt family member 16 (WNT16), estrogen receptor 1 (ESR1), and vitamin D receptor (VDR), have been identified as being associated with OP (13). These two kinds of complex diseases may share some common genetic mechanisms and biological processes. For example, pro-inflammatory cytokines such as TNF, IL-17, IL-6, and IL-1 have been linked to OP (14), and they also play an important role in the development of AS (15).

In addition, many observational studies have demonstrated a close relationship with strong evidence between OP and AS. Vasdev et al. (16–18) found that in patients with AS, the density of the lumbar spine and femoral neck was lower than that of the normal control group. High rates of OP have been documented in patients with AS, ranging from 19% to 62% (19–21). The combination of AS and OP is already an indisputable fact, but the exact mechanism of AS complicated with OP is unclear.

The most commonly cited explanations in the literature include mechanical causes, inflammatory factors, bone metabolic imbalances, and iatrogenic factors (22, 23). Early mechanical reasons proposed by Rubinstein (24) are more widely accepted. Patients with AS may experience limited movement due to pain, morning stiffness, or even bone breaking, resulting in insufficient outdoor exercise and sunshine time, which will undoubtedly lead to wasting by osteoporosis. However, this hypothesis alone is insufficient in explaining the occurrence of OP in the early stages of AS when mobility remains unimpaired. An alternative explanation is an inflammatory hypothesis, which suggests that inflammation triggers both bone resorption and formation in AS (25). The activation of osteoclasts by proinflammatory cytokines, such as interleukin-6 and tumor necrosis factor-alpha, can result in reduced bone mineral density and increased fracture risk (25). Furthermore, there is growing evidence suggesting that genetic factors may also contribute to the development of OP in patients with AS. For instance, certain genetic variations related to bone metabolisms, such as the VDR gene and the receptor activator of the nuclear factor kappa-B ligand (RANKL) gene, have been shown in some studies to potentially affect BMD and fracture risk in patients with AS (26, 27). This genetic hypothesis suggests that identifying such genetic markers could aid in predicting the risk of OP in patients with AS, thus facilitating tailored preventive and therapeutic strategies. However, the complexity of the issue is heightened by the significant differences among individuals with AS. Furthermore, the shared pathophysiological pathways, as well as similar risk factors between AS and OP, make the relationship between these two conditions even more intricate. To better prevent and treat OP in patients with AS, it is necessary to understand the specific mechanism of OP in these patients. Moreover, despite the genetic and physiological links between OP and AS, few studies have examined how OP affects the occurrence and development of AS. Only one report has suggested that OP is a risk factor for AS (28), but whether there is a causal relationship between the two conditions remains unclear.

Therefore, determining whether AS has a direct influence on OP or vice versa will be the primary concern of this study. Due to the bias introduced by confounding factors, the inference of causality from these prior observations is constrained. Randomized controlled trial (RCT) design is the gold standard for determining causality, but it is time-consuming, expensive, and ethically restricted.

Mendelian randomization (MR) is a method for investigating causal relationships and can effectively circumvent the aforementioned limitations by employing genetic variants as exposure instruments (29). Recently, an MR study explored the causality between psoriasis and osteoporosis (30). Similarly, we investigated the genetic relationship between AS and OP using summary data from publicly available genome-wide association studies (GWAS). In the first stage, we examined whether AS has causal effects on BMD measurements. In the second stage, we detected whether BMD measurements are causally associated with AS.

## Methods

### Study design and data sources

**Figure 1:**
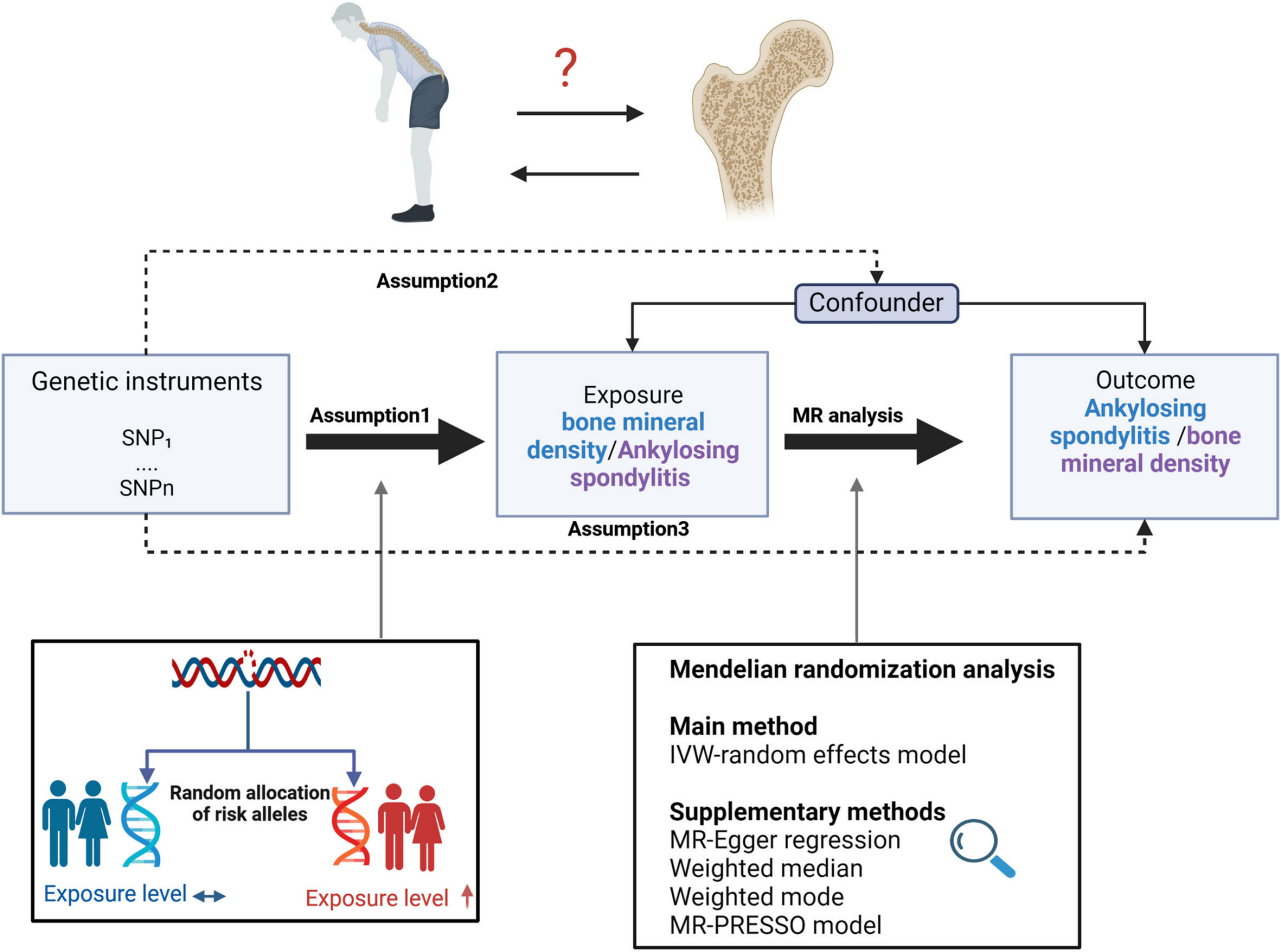
Study design overview and assumptions of the MR design. Assumption 1 indicates that the genetic variants proposed as instrumental variables should be robustly associated with the risk factor of interest, assumption 2 indicates that the used genetic variants should not be associated with potential confounders, and assumption 3 indicates that the selected genetic variants should affect the risk of the outcome merely through the risk factor, not via alternative pathways. MR design can improve causal inference between exposure and outcome by reducing confounding and reverse causality, which are common sources of bias in traditional observational studies. The basis of this is that genetic variants, selected as instrumental variables for studying the effect of modifying the exposure, are randomly allocated at conception and are therefore less vulnerable to confounding from environmental factors and reverse causation. IVW, inverse-variance weighted.

**Figure 1** depicts the study design overview and the MR study’s assumptions. In the analysis of the association between AS and BMD, genetic instruments for the exposure (i.e., AS) were obtained from the IGAS consortium, and the outcomes of the study included BMD by site and age. BMD measured by DXA is widely considered the gold standard for OP diagnosis in clinical practice and is typically assessed at the lumbar spine, forearm, and femoral neck (31). However, regional BMD measurements may be unreliable in skeletally immature children and adolescents. Therefore, whole-body BMD assessment is often employed to examine age-specific OP (32). Furthermore, ultrasound-based heel BMD estimation, while not as standardized as DXA, offers the advantages of a large sample size (due to convenience and affordability) and high heritability (33). In addition, it exhibits a strong correlation with DXA-based BMD (34) and an independent association with fracture risk (35). These factors ensure its reliability as a proxy for OP. Based on the above facts, we selected BMD of five sites, including the total body (TB) BMD, lumbar spine (LS) BMD, femoral neck (FN) BMD, forearm (FA) BMD measured by dual-energy X-ray absorptiometry (DXA), and Heel-BMD by ultrasound, whose datasets were from the GEFOS consortium (FN-BMD, LS-BMD, and FA-BMD), a large GWAS meta-analysis study (TB-BMD), and the UK Biobank (Heel-BMD). Based on the DXA-detected TB-BMD, BMD by age was categorized into five distinct age ranges: 0–15, 15–30, 30–45, 45–60, and over 60. Similarly, in the reverse analysis (i.e. BMD’s effect on AS), we collected the genetic instruments for BMD and treated AS as the outcome, which was defined by the modified New York criteria (36). **Table 1** provides details regarding the data sources used and the demographic profiles of AS and BMD. As this study was based on previously published GWAS summary data, approval from an institutional review board was not necessary, and all participants provided informed consent beforehand.

**Table 1:** Data sources used in this study.

Exposures or outcome	Sample size (total or cases/controls)	Ancestry	Consortia	PubMed ID or URL of original research	URL of available datasets
**Ankylosing spondylitis**	9,069/13,578	European	IGAS Consortium	23749187	https://gwas.mrcieu.ac.uk/datasets/ebi-a-GCST005529/
**Femoral neck bone mineral density**	32735	European	GEFOS Consortium	26367794	https://gwas.mrcieu.ac.uk/datasets/ieu-a-980/
**Lumbar spine bone mineral density**	28498	European	GEFOS Consortium	26367794	https://gwas.mrcieu.ac.uk/datasets/ieu-a-982/
**Forearm bone mineral density**	8143	Mixed	GEFOS Consortium	26367794	https://gwas.mrcieu.ac.uk/datasets/ieu-a-977/
**Heel bone mineral density**	265627	European	UKBiobank	https://data.bris.ac.uk/data/dataset/pnoat8cxo0u52p6ynfaekeigi	https://gwas.mrcieu.ac.uk/datasets/ukb-b-8875/
**Total body bone mineral density**	56284	European	GWAS meta-analysis study	29304378	https://gwas.mrcieu.ac.uk/datasets/ebi-a-GCST005348/
**Total body bone mineral density (age 0-15)**	11807	Mixed (more than 86% European)	GWAS meta-analysis study	29304378	https://gwas.mrcieu.ac.uk/datasets/ebi-a-GCST005345/
**Total body bone mineral density (age 15-30)**	4180	Mixed (more than 86% European)	GWAS meta-analysis study	29304378	https://gwas.mrcieu.ac.uk/datasets/ebi-a-GCST005344/
**Total body bone mineral density (age 30-45)**	10062	Mixed (more than 86% European)	GWAS meta-analysis study	29304378	https://gwas.mrcieu.ac.uk/datasets/ebi-a-GCST005346/
**Total body bone mineral density (age 45-60)**	18805	European	GWAS meta-analysis study	29304378	https://gwas.mrcieu.ac.uk/datasets/ebi-a-GCST005350/
**Total body bone mineral density (age over 60)**	22504	Mixed (more than 86% European)	GWAS meta-analysis study	29304378	https://gwas.mrcieu.ac.uk/datasets/ebi-a-GCST005349/

### Genetic instrument selection

In our bidirectional Mendelian randomization (MR) analysis, we selected genetic instruments based on a consistent standard. To ensure the identification of genetic instrumental variables (IVs) that conformed to the three MR assumptions, we implemented a series of quality control procedures.

First, we employed a genome-wide significance criterion with a threshold of *P*<5E-8 (to mitigate the impact of weak instrument bias) and minor allele frequency (MAF) > 0.01 to identify genetic instruments for AS and BMD. Second, to address the issue of significant linkage disequilibrium (LD), we performed a clumping procedure with R^2^ < 0.001 and a window size of 10,000 kb, utilizing data from the European ancestry-based 1000 Genomes Project (37). In cases where SNP pairs displayed a high LD R^2^ value, the SNP with the lower *P*-value was retained. Third, when the targeted SNPs were not present in the outcome genome-wide association study (GWAS), we sought proxy SNPs that shared high levels of LD (R^2^ > 0.8) with the target SNPs. Finally, to generate a summary set where each SNP in the exposure and outcome corresponded to the same effect allele, we excluded SNPs with discordant alleles and palindromic SNPs by harmonizing the exposure and outcome datasets. These carefully selected SNPs served as the final genetic IVs for our subsequent MR analysis.

Furthermore, in order to assess the reliability and validity of each SNP as a genetic IV in our MR analysis, we calculated the *F*-statistics for each SNP using the equation F=R^2^(N - 2)/(1 - R^2^), where R^2^ represents the proportion of variance in the exposure variable explained by the IV and N represents the sample size of the original GWAS that served as the outcome variable (38). To compute R^2^ for each IV, we utilized the formula R^2^ = (2xEAF(1-EAF)xbeta^2)/[(2xEAF(1-EAF)xbeta^2) + (2xEAF(1-EAF)xNx(SE(beta)^2))], where EAF denotes the effect allele frequency, beta represents the estimated genetic effect on the outcome, N refers to the sample size of the GWAS, and SE stands for the standard error of the genetic effect (39). IVs with *F*-statistics less than 10 were considered unreliable and were excluded from the subsequent MR analysis.

### MR analysis

MR analysis was first conducted to evaluate the causal effect of AS on BMD and then performed in the opposite direction to obtain the causal directionality of the relationship between the two traits.

For the primary MR analysis, we employed the random-effects inverse variance weighted (IVW) technique, which provides a more accurate estimation in the presence of heterogeneity (40). As long as all genetic variants are valid instruments, the IVW can produce unbiased estimates (40). In order to enhance the reliability of our findings, we also applied two additional MR methods: the MR-Egger regression (41) and the median-based estimator, which includes both the weighted median and weighted mode (42). The MR-Egger method has the advantage of being less susceptible to directional pleiotropy and allows for all genetic variants to violate the instrumental variable assumptions. However, the statistical power of MR-Egger is low (43). In contrast, the median-based estimator method is less sensitive to outliers and can provide valid estimates even when up to 50% of the genetic variants are invalid (42). Additionally, when horizontal pleiotropy was detected in certain cases, we applied the MR-PRESSO outlier test to further validate our results, as this method can correct for pleiotropic effects and improve the accuracy of causal estimates (44).

### Heterogeneity, pleiotropy, and sensitivity analysis

To assess horizontal pleiotropy, MR-Egger regression and MR-PRESSO global tests were conducted. The MR-Egger regression’s intercept term indicates the mean pleiotropic effect of the IVs (41). A skewed funnel plot can also suggest the presence of horizontal pleiotropy (40). To detect heterogeneity, we applied Cochrane’s Q statistic and conducted a leave-one-out analysis to check if a single SNP drives the association. In addition, we examined the relationship between the selected SNPs and any possible confounding factors that might affect the association between AS and BMD by searching the PhenoScannerV2 website (http://www.PhenoScanner.medschl.cam.ac.uk/) using linkage disequilibrium (LD) traits (set: *P* for trait-associated SNPs < 5E-8, R^2^ for LD > 0.8 in EUR). Lastly, in the analysis of the impact of AS on BMD, we analyzed the functional information of potential causal genes at the locus of the IVs to confirm that the IVs are not only associated with AS but are also likely to be causative.

### Statistical power calculating

We assessed the statistical power using the mRnd website (https://shiny.cnsgenomics.com/mRnd/) (40). The primary factors of statistical power are the sample size of the outcome and the proportion of variance in the exposure variable explained by the genetic instrument.

### Statistical significance

All statistical analyses were performed using the Two-Sample MR package in R statistical software version 4.2.1. (R Foundation). To account for multiple testing, we considered associations with *P* values below 0.005 (where *P* = 0.05/10) to represent strong evidence of causal associations, and associations with *P*-value below 0.05 but above 0.005 were considered to be suggestive evidence of associations in the MR analysis.

## Results

### Causal effects of AS on BMD by site or at different ages

In our study, 26 independent SNPs were incorporated as instrumental variables (IVs) for AS. However, SNP rs130075 was excluded due to the unavailability of the necessary information for MR tests. Several SNPs were absent from the BMD summary statistic, and some of these were replaced with proxy SNPs, while others were eliminated. Details of the SNP screening process are presented in **Supplementary Table 1**. Each of the final used SNPs had an F-statistic value greater than 10. **Supplementary Table 2** provides specific information regarding IVs for AS. The variance explained by these IVs was approximately 23% for AS.

**Figure 2:**
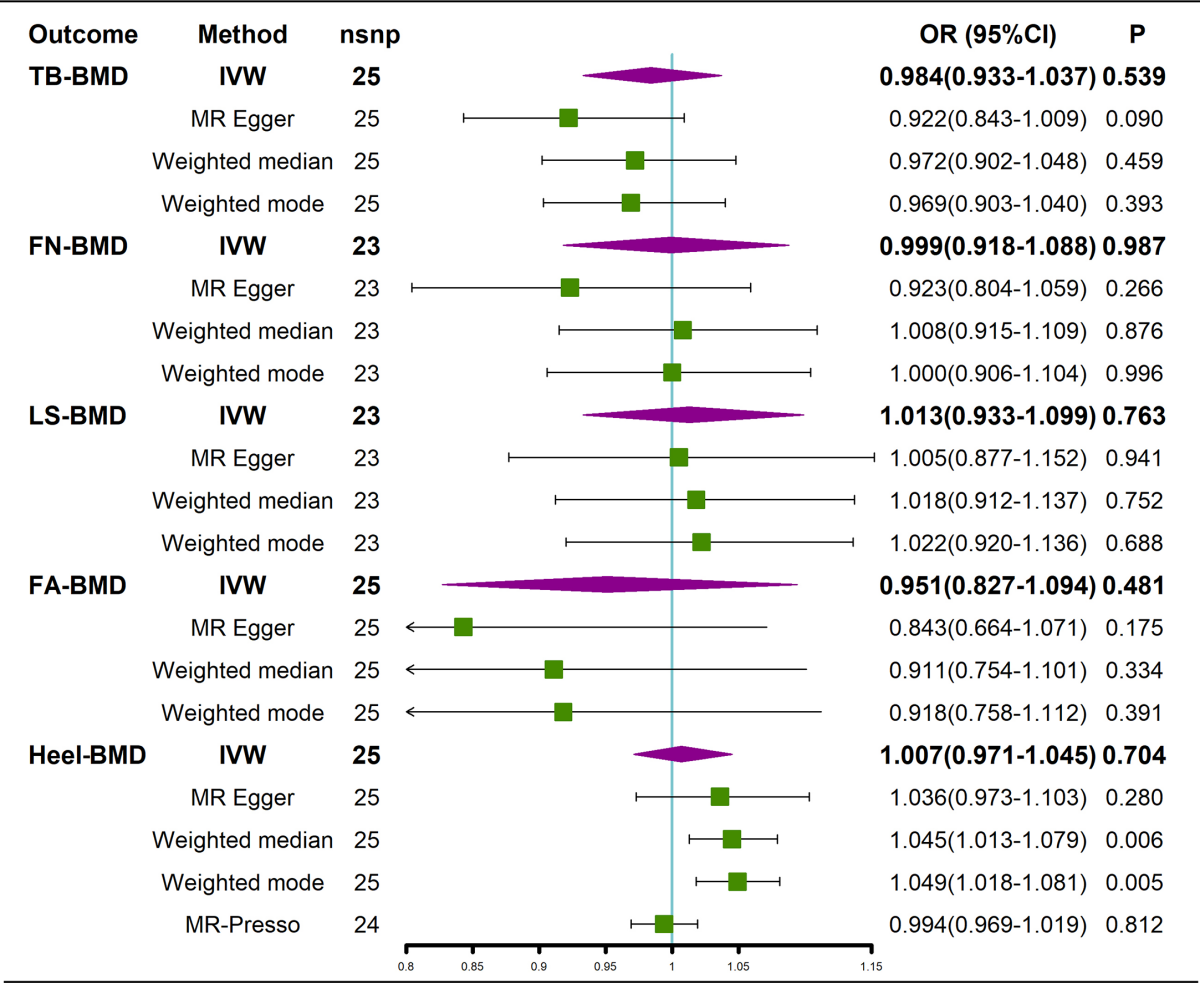
Causal effects of AS on BMD at different sites. AS, Ankylosing spondylitis; FN-BMD, femoral neck bone mineral density; LS-BMD, lumbar spine bone mineral density; TB-BMD, total body bone mineral density; FA-BMD, forearm bone mineral density; Heel-BMD, heel bone mineral density; IVW, inverse variance weighted; nsnp, number of single nucleotide polymorphisms; CI, confidence interval.

**Figure 3:**
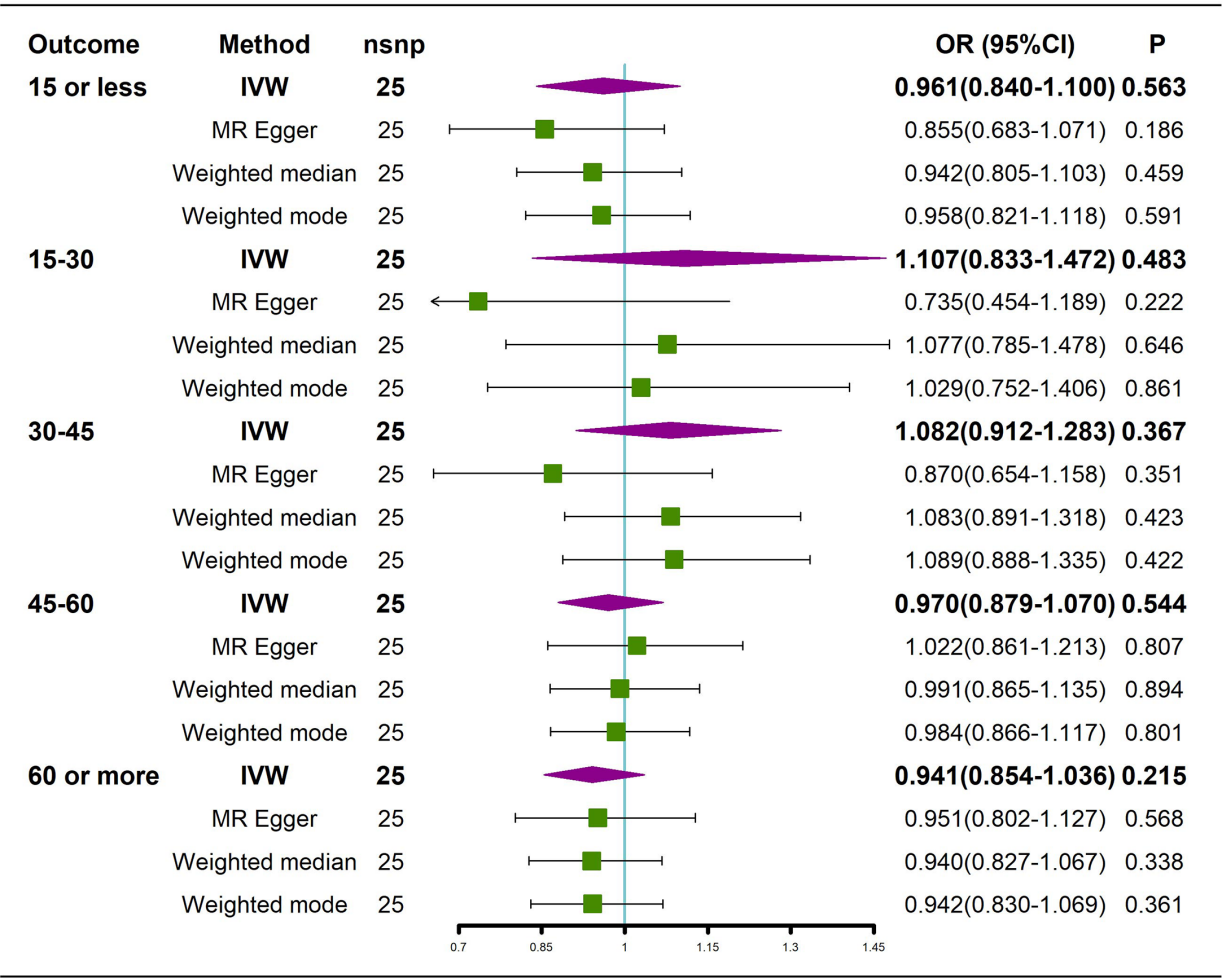
Causal effects of AS on BMD in different age groups. AS: Ankylosing spondylitis; BMD: bone mineral density; IVW, inverse variance weighted; nsnp, number of single nucleotide polymorphisms; CI, confidence interval.

**Figure 4:**
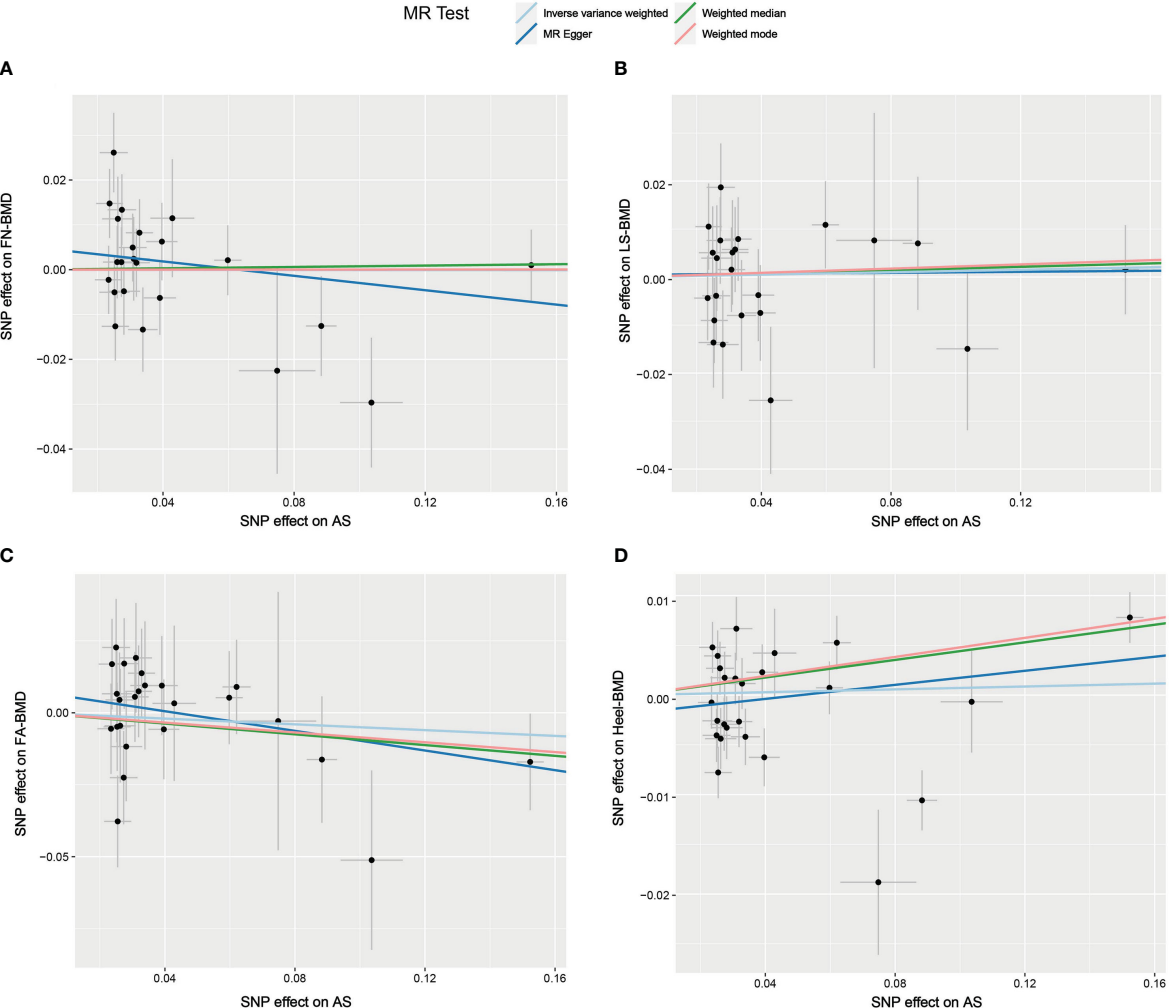
Scatter plot of the causal relationships between AS and BMD at different sites using different MR methods. **(A)** Causal estimates for AS on FN-BMD. **(B)** Causal estimates for AS on LS-BMD **(C)** Causal estimates for AS on FA-BMD. **(D)** Causal estimates for AS on Heel-BMD. The slope of each line corresponds to the causal estimates for each method. Individual SNP effect on the outcome (point and vertical line) against its effect on the exposure (point and horizontal line) is delineated in the background.

**Figure 5:**
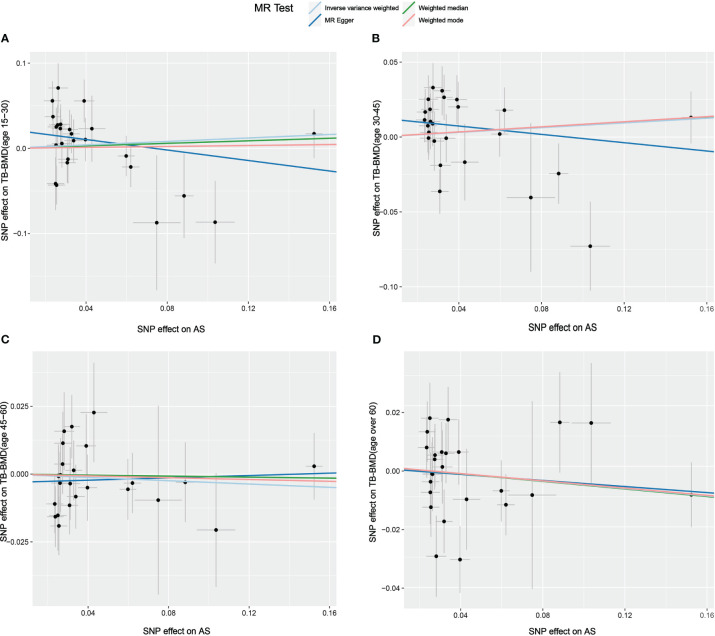
Scatter plot of the causal relationships between AS and BMD in different age groups using different MR methods. **(A)** Causal estimates for AS on TB-BMD (age 15-30). **(B)** Causal estimates for AS on TB-BMD (age 30-45). **(C)** Causal estimates for AS on TB-BMD (age 45-60). **(D)** Causal estimates for AS on TB-BMD (age over 60). The slope of each line corresponds to the causal estimates for each method. Individual SNP effect on the outcome (point and vertical line) against its effect on the exposure (point and horizontal line) is delineated in the background.

The results of the causal analysis of AS on BMD by site or BMD at different ages are presented in **Figures 2** and **3**, respectively. Overall, no significant relationship between genetically predicted AS and decreased BMD levels was found. The main results of IVW showed that there was no statistical link between a higher risk of AS and a lower level of BMD. The same was true for the MR-Egger regression and the median-based estimator (weighted median and weighted mode). Notably, the median-based method showed a causal relationship between genetically predicted AS and an increased level of Heel-BMD (WM: OR 1.045; 95% CI 1.013–1.079; *P* = 0.006), while heterogeneity (Cochrane’s Q in IVW =70.35, *P*=1.93e-06; Cochrane’s Q in MR-Egger =66.88, *P*=3.66e-06, **Table 2**) and horizontal pleiotropy (*P* for MR-Egger intercept 0.29; *P* for MR-PRESSO Global Test 0.001, **Table 2**) may have had an influence on the result (**Table 2**). So we conducted MR-PRESSO to further test the relationship and found no causal effect between them after removing two outlier variants (OR 0.994; 95% CI 0.969–1.019; *P* = 0.812, **Figure 2**). For BMD at other sites and at different ages, there was no heterogeneity between the individual SNPs (**Table 2**). The results of the MR-Egger regression and MR-PRESSO global test suggested that horizontal pleiotropy was unlikely to bias the causation relationship (**Table 2**). A leave-one-out analysis revealed that causal estimates for AS and BMD were not influenced by a single SNP (see **Supplemental Figures 1, 2**). The leave-one-out analysis plots, forest plots, and funnel plots are shown in **Supplementary Figures 1, 2**. The scatter plots for effect sizes of SNPs for AS on BMD by site or at different ages are shown in **Figures 4** and **5**. Further analysis of the functional involvement of potentially causal genes at the locus of IVs revealed that 13 of the 25 instrumental SNPs detected were in or near genes that are functionally linked to AS (see **Supplementary Table 3**). MR analysis by including the 13 casual IVs yielded similar MR results (see **Supplementary Figures 3, 4**).

**Table 2:** MR sensitivity analyses of AS and BMD at different sites and in different age groups.

Exposure	Outcome	No.of Ivs	Heterogeneity tests		Directional horizontal pleiotropy test	
			Methods	Cochran’sQ (*P*)	MR-Egger intercept (*P*)	*P*pleiotropy*
AS	TB-BMD	25	MR Egger	22.02(0.52)	3.89E-03(0.10)	0.468
			IVW	25.01(0.41)		
AS	FN-BMD	23	MR Egger	29.48 (0.10)	5.01E-03(0.17)	0.114
			IVW	32.25(0.07)		
AS	LS-BMD	23	MR Egger	19.76(0.54)	4.67E-04(0.90)	0.666
			IVW	19.78(0.60)		
AS	FA-BMD	25	MR Egger	18.16(0.75)	7.22E-03(0.25)	0.757
			IVW	19.58(0.72)		
AS	Heel-BMD	25	MR Egger	66.88(3.66e-06)	-1.80E-03(0.29)	0.001
			IVW	70.35(1.93e-06)		
AS	15 or less	25	MR Egger	31.73 (0.11)	7.34E-03(0.22)	0.110
			IVW	33.92(0.09)		
AS	15-30	25	MR Egger	33.24(0.08)	2.27E-02(0.06)	0.055
			IVW	39.08(0.027)		
AS	30-45	25	MR Egger	32.69(0.09)	1.29E-02(0.08)	0.059
			IVW	37.38(0.04)		
AS	45-60	25	MR Egger	16.94(0.81)	-3.11E-03(0.47)	0.844
			IVW	17.47(0.83)		
AS	60 or more	25	MR Egger	28.03(0.21)	-6.65E-04(0.88)	0.300
			IVW	28.06(0.26)		
TB-BMD	AS	8	MR Egger	5.17(0.52)	4.27E-03(0.29)	0.155
			IVW	6.49(0.48)		
LS-BMD	AS	5	MR Egger	4.47(0.21)	1.98E-02(0.44)	0.333
			IVW	5.65(0.22)		
Heel-BMD	AS	17	MR Egger	26.12(0.04)	-5.89E-04(0.88)	0.049
			IVW	26.17(0.05)		

* detected by MR-PRESSO Global Test.

To further strengthen our MR assumption, we examined the traits related to our instrumental SNPs. The results of the trait association analysis (see **Supplementary Table 4**) indicated that some SNPs, such as rs1041926, rs4129267, and rs7191548, were associated with certain autoimmune conditions and several potential confounders, such as inflammatory bowel disease, rheumatoid arthritis, levels of C-reactive protein, and frequency of alcohol intake, which may have some impact on OP. Sensitivity analysis by removing these SNPs revealed similar results (see **Supplementary Figures 5, 6**).

### Causal effects of BMD on AS

We incorporated 359, 85, and 24 independent SNPs with a *P*-value of less than 5×10-8 for Heel-BMD, TB-BMD, and LS-BMD, respectively. However, some SNPs were removed based on the aforementioned reasons. Finally, 8 SNPs of TB-BMD, 17 SNPs of Heel-BMD, and 5 SNPs of LS-BMD were used as IVs for the analysis of BMD and the risk of AS. All the final chosen IVs had *F*-statistic values over 10. Detailed information on IVs for BMD is listed in **Supplementary Table 5**. The variation explained by these IVs was 2.1% for TB-BMD, 1.8% for LS-BMD, and 2.2% for Heel-BMD.

**Figure 6:**
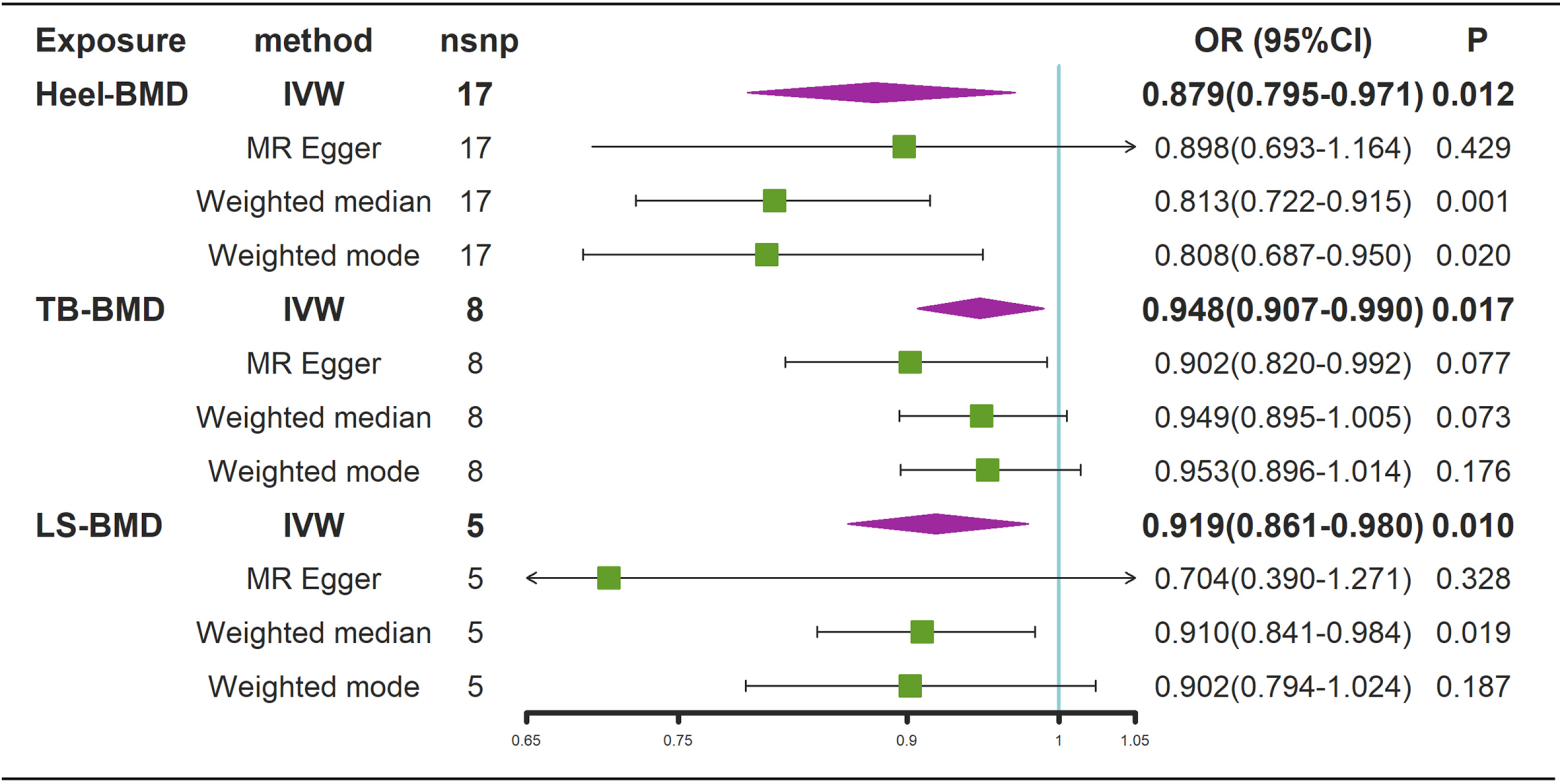
Mendelian randomization analysis results for the effects of BMD on AS. AS, Ankylosing spondylitis; LS-BMD, lumbar spine bone mineral density; TB-BMD, total body bone mineral density; Heel-BMD, heel bone mineral density; IVW, inverse variance weighted; nsnp, number of single nucleotide polymorphisms; CI, confidence interval.

According to the IVW estimator, there were signs of a connection between genetically elevated BMD levels and a decreased risk of AS (Heel-BMD: OR = 0.879, 95% CI: 0.795-0.971, *P* = 0.012; Total-BMD: OR = 0.948, 95% CI: 0.907-0.990, *P* = 0.017; LS-BMD: OR = 0.919, 95% CI: 0.861-0.980, *P* = 0.010, **Figure 6**). The MR-Egger, median-based estimator generated similar findings despite some with lower statistical power. While directional pleiotropy (*P* for MR-Presso Global test = 0.049) and heterogeneity (Cochran’s Q in MR-Egger = 26.12 P = 0.04) were hypothesized in the study of Heel-BMD with AS, there was no indication of heterogeneity or horizontal pleiotropy in the findings of the other MR tests (**Table 2**). The IVW leave-one-out analysis demonstrated that most of the identified relationships were not altered by a single SNP associated with BMD, whereas rs4807630 may have weakened the causal relationship between Heel-BMD and risk of AS (overall *P*-value: 0.011; after removing rs4807630, *P*-value: 6.42 × 10^-4)^. The scatter plot for effect sizes of SNPs for BMD on AS, leave-one-out analysis plots, forest plots, and funnel plots are depicted in **Supplementary Figure 7**.

Traits association analysis (see **Supplementary Table 6**) showed that rs6684375 in Heel-BMD, rs10493013 in TB-BMD, and rs7524102 in LS-BMD are associated with inflammatory bowel disease, which may have some effect on the risk of AS. Sensitivity analysis by removing the SNPs revealed similar results, though with lower statistical power (see **Supplementary Figure 8**).

### Statistical power of the MR analysis

Our MR study produced sufficient statistical power for the analysis of genetically predicted AS with BMD (see **Supplementary Table 7**). We had over 80% power to detect an OR greater than 1.025 or less than 0.976 between AS and TB-BMD, an OR greater than 1.034 or less than 0.967 between AS and FN-BMD, an OR greater than 1.036 or less than 0.965 between AS and LS-BMD, and an OR greater than 1.011 or less than 0.989 between AS and Heel-BMD. Similar results were observed in the analysis of AS on bone mineral density by age (see **Supplementary Table 7**). However, in the analysis of the association between genetically predicted BMD and AS, our MR study yielded less power (see **Supplementary Table 8**). The powers to detect an OR of 0.8 for TB-BMD and AS, LS-BMD and AS, and Heel-BMD and AS were 65%, 58%, and 67%, respectively.

## Discussion

In this study, we used a bidirectional MR method to determine whether genetically predicted AS is causally related to OP or vice versa. Through the largest public GWAS summary data, we failed to detect a causal relationship between genetically elevated AS risk and lower BMD/OP. Associated sensitive analyses proved the reliability of our results. In addition, our study discovered a correlation between genetically increased BMD and a lower risk of AS.

The relationship between AS and OP has been a topic of interest in the medical community, with multiple hypotheses put forth to explain their connection. While OP is already considered one of the common extra-articular manifestations in patients with AS (45), the exact mechanism underlying the coexistence of these conditions is not well understood. Generally, there are two types of contributing factors: intrinsic attributes of AS, including genetic and inflammatory causes, and secondary effects, such as the impact of lower body exercises or medication (e.g. glucocorticoids). The results of our MR study provide evidence in support of the secondary effect hypothesis, which is in line with the early theories proposed by Rubinstein (24). Patients with AS can suffer from significant pain, stiffness, and loss of mobility, which will undoubtedly lead to disuse OP. Besides the mechanical factor, it is not uncommon for patients with AS to have medically induced OP due to prolonged medication use, particularly reduced feeding and impaired nutrient absorption due to gastrointestinal adverse effects associated with prolonged use of NSAIDs (46) and glucocorticoid-related OP (47). Therefore, the secondary effects mechanism of AS-associated OP still needs to be studied in a large sample of clinical data in order to target the prevention and treatment of AS-associated OP. Appropriate exercise can improve BMD, maintain bone structure, and reduce the risk of falls and fragility fractures (48). However, no studies have investigated whether physical activity can improve BMD in patients with AS. Large RCTs will be required in the future to determine the impact of functional exercise on the improvement of OP in patients with AS.

Concerning the “inflammatory hypothesis” of AS merging OP, Grataeos et al. (49) conducted a cohort study with 34 patients, which revealed a significant association between elevated levels of erythrocyte sedimentation rate (ESR) and C-reactive protein (CRP) with OP in patients with AS. Similar results were obtained in another cohort study involving 54 patients by Maillefen et al. (50). However, the conclusions drawn from these studies should be interpreted with caution due to the limited sample size and potential confounding variables. In contrast, Huang et al. (51) conducted an MR study that showed no causal link between high-sensitivity CRP and lower BMD. In addition to ESR and CRP, it has been suggested that a low level of 1,25-dihydroxyvitamin D3 (1,25(OH)2D3) may contribute to OP in patients with AS by inhibiting osteogenesis and reducing osteoblast activity (52). However, an MR study by Tang Yanchao et al. (53) did not find a causal relationship between genetically determined vitamin D levels and BMD. Nevertheless, given the limited statistical power of the aforementioned MR study, further research is warranted to explore the potential inflammatory mechanisms underlying the development of OP in patients with AS.

Apart from the possible inflammatory mechanisms, the contribution of genetics to the comorbidity of AS and OP is also a subject of interest in this topic. The heritability of AS has been estimated to be approximately 32.7% (54), while the heritability of OP has been estimated to be as high as 50-85% (55). This suggests a significant genetic component to both conditions. Currently, GWAS have identified many genetic markers for OP and have explained approximately 5% of its heritability (56). GWAS analysis of AS has identified multiple susceptible sites, including HLA-B27, which has explained approximately 28% of the heritability of AS (54). In our study, 24 SNPs were used as the IVs that explained more than 20% of the heritability of AS. Through this high statistical power, our MR study failed to detect a causal relationship between genetically predicted AS and OP. Nevertheless, this does not mean that the genetic effects of AS do not influence the risk of concomitant OP. Because of the intricate and multifaceted impact of AS on bone health, AS patients may concurrently experience bone formation and bone resorption. This may, to some extent, hinder the identification of a clear causal link between AS and OP.

In clinical practice, AS is closely associated with OP. This association may be driven not only by the secondary effects of AS but also by the common pathogenesis or metabolic interaction of the two conditions. Our study revealed that these two conditions share many common genetic loci, mainly distributed in genes such as IL23R, RUNX3, LRP5, TBKBP1, and WNT16 (see **Supplementary Table 9**). These genes primarily participate in signaling pathways such as JAK-Stat, TGF-β, Wnt, and TNF, which are known to be involved in both AS and OP. The Wnt signaling pathway is a critical regulator of bone formation, homeostasis, inflammation, and immune responses (57). Moreover, in the context of AS, this pathway has received significant attention due to its reported dysregulation in the pathogenesis of AS, which may further exacerbate the disease’s progression (57, 58). Specifically, elevated expression levels of Wnt proteins have been found in the spinal tissues and serum of patients with AS when compared to healthy controls (58). The intensity of inflammation and cytokines, such as TNF, which is elevated in AS, also influences the Wnt signaling pathway (58). Additionally, this pathway may mediate the effects of inflammation on bone formation and immune suppression in AS by activating downstream pathways such as mTORC1, PD-L1, and PKCδ (58). The Wnt signaling pathway also plays an important role in OP (59), which promotes osteoblast differentiation and function while inhibiting osteoclast development and activity, thereby stimulating bone formation (59, 60). Furthermore, the Wnt signaling pathway is activated by the binding of Wnt ligands to receptors on the cell surface, such as LRP5 and LRP6 (59, 60), and previous studies have shown that mutations or functional variations in these receptors can lead to low or high human bone density phenotypes (61). The Transforming growth factor-beta (TGF-β) signaling pathway is also a hot topic in research due to its involvement in regulating various physiological processes, such as inflammation, differentiation, and fibrosis (62). TGF-β is closely related to the pathogenesis of AS (63–65) and multiple studies have shown that the level of TGF-β in the serum of patients with AS is significantly elevated, regardless of disease activity level (65–68). TGF-β could potentially facilitate the development of AS by triggering osteoblast differentiation and bone formation, augmenting fibroblast proliferation and collagen synthesis, as well as regulating immune responses and cytokine production (67, 69, 70). TGF-β also plays a role in OP (71) by promoting osteoblast differentiation and inhibiting osteoclast development, which can stimulate bone formation (72). Nevertheless, it’s worth noting that TGF-β may also have detrimental effects on bone quality and strength. Specifically, it can induce excessive collagen synthesis and cross-linking, impair bone mineralization, and decrease the bone turnover rate (72). Inflammation and mechanical stress in AS can dysregulate Wnt and TGF-β pathways, leading to low bone density and impaired bone formation. In addition to TGF-β and Wnt signaling pathways, polymorphisms in vitamin D receptor, estrogen receptor, type Iα1 collagen, and osteoprotegerin genes are also associated with OP and AS (25). Further research is needed to elucidate their exact roles and mechanisms in the relationship between the two.

Considering the shared pathogenic mechanisms between AS and OP, it is important to investigate the potential cross-effects of drug treatments for these diseases. Additionally, since the mechanism of OP in patients with AS varies due to individual differences, the proportion of different factors (physical activity, genetic factors, and inflammation-related factors) in the pathogenesis is also different. Therefore, it is worth exploring whether there are targeted drug treatments for different AS patients with OP instead of using a one-size-fits-all treatment model.

Currently, it is unclear whether medications used to treat AS are effective in treating OP. One such medication is tumor necrosis factor-alpha (TNF-α) inhibitors. These drugs work by blocking the binding of TNF-α to its receptor, which can help balance bone metabolism and reduce inflammation (73, 74). The efficacy of TNF-α inhibitors in treating patients with AS has been validated in clinical practice, including improvements in joint function, pain relief, reduced BASDAI scores, and lower CRP levels (75). However, their effectiveness in treating OP is still uncertain. Some studies suggest that TNF-α inhibitors may help increase bone density and lower bone turnover markers, which could prevent or treat OP (76). However, other studies have found no significant effects or even negative consequences on OP (77). IL-17A inhibitors, as biological agents, are also frequently utilized in the management of AS. However, their potential efficacy in treating OP remains inconclusive. A review article critically evaluated the intricacies of IL-17A signaling in bone remodeling, postulating that IL-17A exhibits both positive and negative effects on the process. Therefore, further research is needed to determine the effectiveness of IL-17A in treating OP (78).

Studies investigating the impact of OP medications on AS have predominantly focused on bisphosphonates. Bisphosphonates are commonly used to treat OP by preventing bone resorption and reducing the risk of fractures. Recent studies have suggested that bisphosphonates may also have anti-inflammatory benefits for patients with AS (79). A review article noted that bisphosphonates can reduce joint damage in autoimmune arthritis by regulating the generation of pro-inflammatory cytokines and affecting T and B cell function, in addition to their anti-resorptive effects (80). However, the effectiveness and safety of bisphosphonates in treating AS is still uncertain. While an RCT found that intravenous injection of bisphosphonates improved clinical and laboratory indicators in patients with AS, including reducing levels of CRP, erythrocyte sedimentation rate, and serum amyloid A (79), another meta-analysis did not find significant differences between patients with AS treated with bisphosphonates and those who did not use the drug, except for follow-up ESR (81). Aside from their potential anti-inflammatory effects, bisphosphonates may also affect bone formation and repair in patients with AS, but the evidence is insufficient. For example, a study found that alkaline phosphatase is highly expressed in patients with AS, which may contribute to stiffness and could be a potential therapeutic target (82). Research has suggested that bisphosphonates can inhibit alkaline phosphatase, which may reduce stiffness in patients with AS through this pathway. Other OP drugs, such as selective estrogen receptor modulators (SERMs), calcitonin, and methylnaltrexone, may also have non-typical effects on AS treatment beyond changes in bone density. Further research is necessary to determine the most tailored drug regimen for AS patients with concurrent OP.

While our study did not identify a significant impact of genetic liability to AS on the OP, we did observe a potential link between the genetic prediction of changes in BMD and susceptibility to AS. The impact of changes in BMD on AS suggests the involvement of bone remodeling mechanisms in the pathogenesis of AS, implying that OP in patients with AS may not only be a secondary phenomenon caused by inflammation and movement disorders but also a primary manifestation of AS (25). The causes of OP in patients with AS may vary from person to person. While Rubinstein’s early mechanical theory was widely accepted, it fails to explain the development of OP in the early stages of AS (18, 83), leading to the emergence of the inflammatory hypothesis. Our study suggests that the development of OP in patients with AS in the early stages may not be secondary to inflammation, but instead, may occur alongside AS due to the impact of bone metabolism-associated mechanisms on AS development. For instance, the gene WNT16, in which the BMD-associated SNP rs3801387 is located (see **Supplementary Table 9**), has been proven to regulate the formation of the spine and muscles through signals from the notochord and dermomyotome (84), which may have an effect on AS. Besides, from a clinical point of view, our results show that OP is a risk factor for AS. Though clinical studies on the effect of BMD on the development of AS are rare, one article on screening risk factors for autoimmune arthritis (including AS) found that OP was a risk factor for the development of AS (OR 2.93, 95% CI 2.00-4.29) (28), which is consistent with our findings. This implies that individuals diagnosed with OP, especially those who are youthful and have a familial history of hereditary OP, should be made aware of the potential risk of developing AS. The application of polygenic risk scores (PRSs) could serve as a promising approach to aid in the identification of AS in this population (85). Furthermore, additional clinical research is required to determine whether screening for BMD in young patients can aid in the early diagnosis of AS. A study compared the clinical characteristics of AS in individuals with early-onset (onset age <50 years) and late-onset (onset age ≥50 years) and found that late-onset AS has different clinical characteristics compared to early-onset AS, suggesting that these groups may have distinct underlying causes or mechanisms of the disease (86). Based on this finding and our results, we believe that genetic and environmental factors related to bone metabolism and inflammation may interact to cause the emergence of late-onset AS after the onset of senile osteoporosis. Therefore, we suggest that elderly patients with OP should also be aware of the risk of having AS.

The major strengths of this research work include large-scale GWAS cases, detailed analyses of IV selection, and multiple methods used to obtain a robust result. To our knowledge, our study has the largest sample size to date of any study examining the relationship between AS and BMD. However, our study also has some limitations. First, due to sample limitations, this study did not stratify early-stage patients from late-stage patients. High BMD measured by DXA in late-stage AS patients due to ossification of several areas of the spine (e.g., ligamentous tuberosity, vertebral ligament ossification, small joint fusion, etc.) may have introduced some bias, but we compensated for this by using BMD measured by multiple methods and BMD at different site and age. In addition, DXA can only measure surface area BMD and is insufficiently sensitive; the absence of a decrease in surface area BMD does not rule out local to-point bone loss. Compared to DXA, QCT measures true volumetric bone density in mg/cm3, which is a more sensitive reflection of BMD changes in osteoporosis (87, 88). Further studies are still needed in the future using data from QCT. Second, the current study did not distinguish between the sex of the patients, but it is good to note that the original study was adjusted for sex. Third, the present study contains only summary data and no specific clinical data, such as activity level or medication use, so future research will have to examine the mediating effects of activity level and medication use on the development of osteoporosis in AS and their proportion. Finally, the population of this study is mainly European, so the effect on other ethnic groups is unknown.

In conclusion, this MR study found that the causal association between genetic liability to AS and the risk of OP or lower BMD in the European population was not evident, which highlights the second effect (e.g., mechanical reasons such as limited movement) of AS on OP. Functional exercise might be an effective strategy to treat and prevent OP in patients with AS. Simultaneously, our study revealed a causal relationship between genetically predicted decreased BMD/OP and AS, indicating that OP is a significant risk factor for AS. Therefore, patients with OP should be vigilant about the possibility of developing AS. These findings also suggest the potential for developing new diagnostic and therapeutic strategies for AS through the exploration of OP-related pathways. Additionally, the shared pathogenesis and pathways of OP and AS suggest that there may be potential drug interactions between treatments for both diseases. Further research is needed to determine whether more optimized and individualized treatment regimens for patients with concurrent osteoporosis and ankylosing spondylitis can be developed.

## Data availability statement

The original contributions presented in the study are included in the article/**Supplementary Material**. Further inquiries can be directed to the corresponding authors.

## Ethics statement

Ethical review and approval was not required for the study on human participants in accordance with the local legislation and institutional requirements. Written informed consent for participation was not required for this study in accordance with the national legislation and the institutional requirements.

## Author contributions

JM and HH conceived the presented idea. JM and HH developed the theory and performed the computations. HD, YH and WZ verified the analytical methods. JM and HH drafted the manuscript. XC and XF reviewed the manuscript. All authors discussed the results and contributed to the final manuscript.

## References

[1] Braun J Sieper J. Ankylosing spondylitis. Lancet (2007) 369:1379–90. doi: 10.1016/s0140-6736(07)60635-7 17448825

[2] Crossfield SSR Marzo-Ortega H Kingsbury SR Pujades-Rodriguez M Conaghan PG. Changes in ankylosing spondylitis incidence, prevalence and time to diagnosis over two decades. RMD Open (2021) 7:e001888. doi: 10.1136/rmdopen-2021-001888 34887345 PMC8663075

[3] Haroon NN Paterson JM Li P Haroon N. Increasing proportion of female patients with ankylosing spondylitis: a population-based study of trends in the incidence and prevalence of AS. BMJ Open (2014) 4:e006634. doi: 10.1136/bmjopen-2014-006634 PMC4267076 25510888

[4] Dean LE Jones GT MacDonald AG Downham C Sturrock RD Macfarlane GJ. Global prevalence of ankylosing spondylitis. Rheumatology (2013) 53:650–7. doi: 10.1093/rheumatology/ket387 24324212

[5] Park J-S Hong J-Y Park Y-S Han K Suh S-W. Trends in the prevalence and incidence of ankylosing spondylitis in south Korea, 2010–2015 and estimated differences according to income status. Sci Rep (2018) 8:7694. doi: 10.1038/s41598-018-25933-4 29769560 PMC5955990

[6] Anam AK Insogna K. Update on osteoporosis screening and management. Med Clin North Am (2021) 105:1117–34. doi: 10.1016/j.mcna.2021.05.016 34688418

[7] Reginster J-Y Burlet N. Osteoporosis: a still increasing prevalence. Bone (2006) 38:4–9. doi: 10.1016/j.bone.2005.11.024 16455317

[8] Lane NE. Epidemiology, etiology, and diagnosis of osteoporosis. Am J Obstet Gynecol (2006) 194:S3–S11. doi: 10.1016/j.ajog.2005.08.047 16448873

[9] Haroon N. Endoplasmic reticulum aminopeptidase 1 and interleukin-23 receptor in ankylosing spondylitis. Curr Rheumatol Rep (2012) 14:383–9. doi: 10.1007/s11926-012-0268-0 22782541

[10] Xia Y Liu Y-Q Chen K Wang L-C Ma C-Y Zhao Y-R. Association of IL-1R2 genetic polymorphisms with the susceptibility of ankylosing spondylitis in northern Chinese han population. Mod Rheumatol (2015) 25:908–12. doi: 10.3109/14397595.2015.1024302 25736356

[11] Davidson SI Liu Y Danoy PA Wu X Thomas GP Jiang L. Association of STAT3 and TNFRSF1A with ankylosing spondylitis in han Chinese. Ann Rheum Dis (2010) 70:289–92. doi: 10.1136/ard.2010.133322 21068102

[12] Consortium IG of AS. Identification of multiple risk variants for ankylosing spondylitis through high-density genotyping of immune-related loci. Nat Genet (2013) 45:730–8. doi: 10.1038/ng.2667 PMC3757343 23749187

[13] Clark GR Duncan EL. The genetics of osteoporosis. Br Med Bull (2015) 113:73–81. doi: 10.1093/bmb/ldu042 25634850

[14] Cagnetta V Patella V. The role of the immune system in the physiopathology of osteoporosis. Clin Cases Miner Bone Metab (2012) 9:85–8. PMC3476525 23087716

[15] Ranganathan V Gracey E Brown MA Inman RD Haroon N. Pathogenesis of ankylosing spondylitis [[/amp]]mdash; recent advances and future directions. Nat Rev Rheumatol (2017) 13:359–67. doi: 10.1038/nrrheum.2017.56 28446810

[16] Toussirot E. Bone density, ultrasound measurements and body composition in early ankylosing spondylitis. Rheumatology (2001) 40:882–8. doi: 10.1093/rheumatology/40.8.882 11511757

[17] Muntean L Rojas-Vargas M Font P Simon S-P Rednic S Schiotis R. Relative value of the lumbar spine and hip bone mineral density and bone turnover markers in men with ankylosing spondylitis. Clin Rheumatol (2011) 30:691–5. doi: 10.1007/s10067-010-1648-3 21221691

[18] Vasdev V Bhakuni D Garg MK Narayanan K Jain R Chadha D. Bone mineral density in young males with ankylosing spondylitis. Int J Rheum Dis (2010) 14:68–73. doi: 10.1111/j.1756-185x.2010.01577.x 21303484

[19] Dubrovsky AM Lim MJ Lane NE. Osteoporosis in rheumatic diseases: anti-rheumatic drugs and the skeleton. Calcif Tissue Int (2018) 102:607–18. doi: 10.1007/s00223-018-0401-9 29470611

[20] van der Weijden MAC Claushuis TAM Nazari T Lems WF Dijkmans BAC van der Horst-Bruinsma IE. High prevalence of low bone mineral density in patients within 10 years of onset of ankylosing spondylitis: a systematic review. Clin Rheumatol (2012) 31:1529–35. doi: 10.1007/s10067-012-2018-0 PMC3483100 22706444

[21] Davey-Ranasinghe N Deodhar A. Osteoporosis and vertebral fractures in ankylosing spondylitis. Curr Opin Rheumatol (2013) 25:509–16. doi: 10.1097/bor.0b013e3283620777 23719363

[22] Castañeda S Garcés-Puentes M Bernad Pineda M. Fisiopatología de la osteoporosis en las enfermedades articulares inflamatorias crónicas. Rev Osteoporos Metab Miner (2021) 13:32–8. doi: 10.4321/s1889-836x2021000100006

[23] Singh HJ Nimarpreet K Ashima Das S Kumar A Prakash S. Study of bone mineral density in patients with ankylosing spondylitis. J Clin Diagn Res (2013) 7:2832–5. doi: 10.7860/JCDR/2013/6779.3770 PMC3919376 24551650

[24] Rubinstein HM. Osteoporosis in ankylosing spondylitis. Rheumatology (1991) 30:160–0. doi: 10.1093/rheumatology/30.2.160 2012957

[25] Magrey M Khan MA. Osteoporosis in ankylosing spondylitis. Curr Rheumatol Rep (2010) 12:332–6. doi: 10.1007/s11926-010-0122-1 20680529

[26] Lange U Teichmann J Obermayer-Pietsch B. Genetische aspekte zur knochendichteminderung bei ankylosierender spondylitis. Z Orthop Unfall (2009) 147:577–81. doi: 10.1055/s-0029-1185711 19938354

[27] Wang C-M Tsai S-C Lin J-C Wu Y-JJ Wu J Chen J-Y. Association of genetic variants of RANK, RANKL, and OPG with ankylosing spondylitis clinical features in Taiwanese. Mediators Inflammation (2019) 2019:1–14. doi: 10.1155/2019/8029863 PMC6446096 31015798

[28] Meer E Thrastardottir T Wang X Dubreuil M Chen Y Gelfand JM. Risk factors for diagnosis of psoriatic arthritis, psoriasis, rheumatoid arthritis, and ankylosing spondylitis: a set of parallel case-control studies. J Rheumatol (2021) 49:53–9. doi: 10.3899/jrheum.210006 34334358

[29] O’Donnell CJ Sabatine MS. Opportunities and challenges in mendelian randomization studies to guide trial design. JAMA Cardiol (2018) 3:967. doi: 10.1001/jamacardio.2018.2863 30326490

[30] Xia J Xie S-Y Liu K-Q Xu L Zhao P-P Gai S-R. Systemic evaluation of the relationship between psoriasis, psoriatic arthritis and osteoporosis: observational and mendelian randomisation study. Ann Rheum Dis (2020) 79:1460–7. doi: 10.1136/annrheumdis-2020-217892 PMC7970448 32737104

[31] Lewiecki EM Binkley N Morgan SL Shuhart CR Camargos BM Carey JJ. Best practices for dual-energy X-ray absorptiometry measurement and reporting: international society for clinical densitometry guidance. J Clin Densitom (2016) 19:127–40. doi: 10.1016/j.jocd.2016.03.003 27020004

[32] Medina-Gomez C Kemp JP Trajanoska K Luan J Chesi A Ahluwalia TS. Life-course genome-wide association study meta-analysis of total body BMD and assessment of age-specific effects. Am J Hum Genet (2018) 102:88–102. doi: 10.1016/j.ajhg.2017.12.005 29304378 PMC5777980

[33] Howard GM Nguyen TV Harris M Kelly PJ Eisman JA. Genetic and environmental contributions to the association between quantitative ultrasound and bone mineral density measurements: a twin study. J Bone Miner Res (1998) 13:1318–27. doi: 10.1359/jbmr.1998.13.8.1318 9718201

[34] Gonnelli S Cepollaro C Gennari L Montagnani A Caffarelli C Merlotti D. Quantitative ultrasound and dual-energy X-ray absorptiometry in the prediction of fragility fracture in men. Osteoporos Int (2004) 16:963–8. doi: 10.1007/s00198-004-1771-6 15599495

[35] Bauer DC Ewing SK Cauley JA Ensrud KE Cummings SR Orwoll ES. Quantitative ultrasound predicts hip and non-spine fracture in men: the MrOS study. Osteoporos Int (2007) 18:771–7. doi: 10.1007/s00198-006-0317-5 17273893

[36] Linden SVD Valkenburg HA Cats A. Evaluation of diagnostic criteria for ankylosing spondylitis. Arthritis Rheum (1984) 27:361–8. doi: 10.1002/art.1780270401 6231933

[37] Consortium T 1000 GP. A map of human genome variation from population-scale sequencing. Nature (2010) 467:1061–73. doi: 10.1038/nature09534 PMC3042601 20981092

[38] Burgess S Thompson SG Collaboration CCG. Avoiding bias from weak instruments in mendelian randomization studies. Int J Epidemiol (2011) 40:755–64. doi: 10.1093/ije/dyr036 21414999

[39] Papadimitriou N Dimou N Tsilidis KK Banbury B Martin RM Lewis SJ. Physical activity and risks of breast and colorectal cancer: a mendelian randomisation analysis. Nat Commun (2020) 11:597. doi: 10.1038/s41467-020-14389-8 32001714 PMC6992637

[40] Hemani G Zheng J Elsworth B Wade KH Haberland V Baird D. The MR-base platform supports systematic causal inference across the human phenome. eLife (2018) 7:e34408. doi: 10.7554/elife.34408 29846171 PMC5976434

[41] Bowden J Davey Smith G Burgess S. Mendelian randomization with invalid instruments: effect estimation and bias detection through egger regression. Int J Epidemiol (2015) 44:512–25. doi: 10.1093/ije/dyv080 PMC4469799 26050253

[42] Bowden J Davey Smith G Haycock PC Burgess S. Consistent estimation in mendelian randomization with some invalid instruments using a weighted median estimator. Genet Epidemiol (2016) 40:304–14. doi: 10.1002/gepi.21965 PMC4849733 27061298

[43] Burgess S Thompson SG. Interpreting findings from mendelian randomization using the MR-egger method. Eur J Epidemiol (2017) 32:377–89. doi: 10.1007/s10654-017-0255-x PMC5506233 28527048

[44] Verbanck M Chen C-Y Neale B Do R. Detection of widespread horizontal pleiotropy in causal relationships inferred from mendelian randomization between complex traits and diseases. Nat Genet (2018) 50:693–8. doi: 10.1038/s41588-018-0099-7 PMC6083837 29686387

[45] Ma KS Lee Y Lin C Shih P Wei JC. Management of extra-articular manifestations in spondyloarthritis. Int J Rheum Dis (2023) 26:183–6. doi: 10.1111/1756-185x.14485 36703270

[46] Vestergaard P Hermann P Jensen J-EB Eiken P Mosekilde L. Effects of paracetamol, non-steroidal anti-inflammatory drugs, acetylsalicylic acid, and opioids on bone mineral density and risk of fracture: results of the Danish osteoporosis prevention study (DOPS). Osteoporos Int (2011) 23:1255–65. doi: 10.1007/s00198-011-1692-0 21710339

[47] Canalis E Mazziotti G Giustina A Bilezikian JP. Glucocorticoid-induced osteoporosis: pathophysiology and therapy. Osteoporos Int (2007) 18:1319–28. doi: 10.1007/s00198-007-0394-0 17566815

[48] Santos L Elliott-Sale KJ Sale C. Exercise and bone health across the lifespan. Biogerontology (2017) 18:931–46. doi: 10.1007/s10522-017-9732-6 PMC5684300 29052784

[49] Gratacós J Collado A Pons F Osaba M Sanmartí R Roqué M. Significant loss of bone mass in patients with early, active ankylosing spondylitis: a followup study. Arthritis Rheum (1999) 42:2319–24. doi: 10.1002/1529-0131(199911)42:11<2319::AID-ANR9>3.0.CO;2-G 10555026

[50] Maillefert JF Aho LS El Maghraoui A Dougados M Roux C. Changes in bone density in patients with ankylosing spondylitis: a two-year follow-up study. Osteoporos Int (2001) 12:605–9. doi: 10.1007/s001980170084 11527060

[51] Huang JV Schooling CM. Inflammation and bone mineral density: a mendelian randomization study. Sci Rep (2017) 7:8666. doi: 10.1038/s41598-017-09080-w 28819125 PMC5561220

[52] Lange U Teichmann J Strunk J Müller-Ladner U Schmidt KL. Association of 1.25 vitamin D3 deficiency, disease activity and low bone mass in ankylosing spondylitis. Osteoporos Int (2005) 16:1999–2004. doi: 10.1007/s00198-005-1990-5 16172800

[53] Tang Y Wei F Yu M Zhou H Wang Y Cui Z. Absence of causal association between vitamin d and bone mineral density across the lifespan: a mendelian randomization study. Sci Rep (2022) 12:10408. doi: 10.1038/s41598-022-14548-5 35729194 PMC9213555

[54] Ellinghaus D Consortium TIIG Jostins L Spain SL Cortes A Bethune J. Analysis of five chronic inflammatory diseases identifies 27 new associations and highlights disease-specific patterns at shared loci. Nat Genet (2016) 48:510–8. doi: 10.1038/ng.3528 PMC4848113 26974007

[55] Ioannidis JP Ng MY Sham PC Zintzaras E Lewis CM Deng H-W. Meta-analysis of genome-wide scans provides evidence for sex- and site-specific regulation of bone mass. J Bone Miner Res (2006) 22:173–83. doi: 10.1359/jbmr.060806 PMC4016811 17228994

[56] Richards JB Zheng H-F Spector TD. Genetics of osteoporosis from genome-wide association studies: advances and challenges. Nat Rev Genet (2012) 13:576–88. doi: 10.1038/nrg3228 22805710

[57] Corr M. Wnt signaling in ankylosing spondylitis. Clin Rheumatol (2014) 33:759–62. doi: 10.1007/s10067-014-2663-6 24820146

[58] Li X Wang J Zhan Z Li S Zheng Z Wang T. Inflammation intensity-dependent expression of osteoinductive wnt proteins is critical for ectopic new bone formation in ankylosing spondylitis. Arthritis Rheumatol (2018) 70:1056–70. doi: 10.1002/art.40468 29481736

[59] Amjadi-Moheb F Akhavan-Niaki H. Wnt signaling pathway in osteoporosis: epigenetic regulation, interaction with other signaling pathways, and therapeutic promises. J Cell Physiol (2019) 234:14641–50. doi: 10.1002/jcp.28207 30693508

[60] Canalis E. Wnt signalling in osteoporosis: mechanisms and novel therapeutic approaches. Nat Rev Endocrinol (2013) 9:575–83. doi: 10.1038/nrendo.2013.154 23938284

[61] Baron R Kneissel M. WNT signaling in bone homeostasis and disease: from human mutations to treatments. Nat Med (2013) 19:179–92. doi: 10.1038/nm.3074 23389618

[62] Clark DA Coker R. Molecules in focus transforming growth factor-beta (TGF-β). Int J Biochem Cell Biol (1998) 30:293–8. doi: 10.1016/s1357-2725(97)00128-3 9611771

[63] van der Paardt M. Susceptibility to ankylosing spondylitis: no evidence for the involvement of transforming growth factor 1 (TGFB1) gene polymorphisms. Ann Rheum Dis (2005) 64:616–9. doi: 10.1136/ard.2004.027698 PMC1755451 15769917

[64] Xu S Zhang X Ma Y Chen Y Xie H Yu L. FOXO3a alleviates the inflammation and oxidative stress via regulating TGF-β and HO-1 in ankylosing spondylitis. Front Immunol (2022) 13:935534. doi: 10.3389/fimmu.2022.935534 35784335 PMC9247177

[65] Ding L Yin Y Hou Y Jiang H Zhang J Dai Z. microRNA-214-3p suppresses ankylosing spondylitis fibroblast osteogenesis via BMP–TGFβ axis and BMP2. Front Endocrinol (2021) 11:609753. doi: 10.3389/fendo.2020.609753 PMC8082363 33935961

[66] Taylan A Sari I Kozaci DL Yuksel A Bilge S Yildiz Y. Evaluation of the T helper 17 axis in ankylosing spondylitis. Rheumatol Int (2011) 32:2511–5. doi: 10.1007/s00296-011-1995-7 21833527

[67] Yu T Zhang J Zhu W Wang X Bai Y Feng B. Chondrogenesis mediates progression of ankylosing spondylitis through heterotopic ossification. Bone Res (2021) 9:19. doi: 10.1038/s41413-021-00140-6 33731675 PMC7969928

[68] Tang Y Wu X Lei W Pang L Wan C Shi Z. TGF-β1–induced migration of bone mesenchymal stem cells couples bone resorption with formation. Nat Med (2009) 15:757–65. doi: 10.1038/nm.1979 PMC2727637 19584867

[69] Sanjabi S Oh SA Li MO. Regulation of the immune response by TGF-β: from conception to autoimmunity and infection. Cold Spring Harb Perspect Biol (2017) 9:a022236. doi: 10.1101/cshperspect.a022236 28108486 PMC5453394

[70] Erlebacher A Derynck R. Increased expression of TGF-beta 2 in osteoblasts results in an osteoporosis-like phenotype. J Cell Biol (1996) 132:195–210. doi: 10.1083/jcb.132.1.195 8567723 PMC2120709

[71] Tu M-Y Han K-Y Lan Y-W Chang K-Y Lai C-W Staniczek T. Association of TGF-β1 and IL-10 gene polymorphisms with osteoporosis in a study of Taiwanese osteoporotic patients. Genes (2021) 12:930. doi: 10.3390/genes12060930 34207210 PMC8233820

[72] Wu M Chen G Li Y-P. TGF-β and BMP signaling in osteoblast, skeletal development, and bone formation, homeostasis and disease. Bone Res (2016) 4:16009. doi: 10.1038/boneres.2016.9 27563484 PMC4985055

[73] Siderius M Spoorenberg A Kroese FGM van der Veer E Arends S. After an initial balance favoring collagen formation and mineralization, bone turnover markers return to pre-treatment levels during long-term TNF-α inhibition in patients with ankylosing spondylitis. PloS One (2023) 18:e0283579. doi: 10.1371/journal.pone.0283579 36961859 PMC10038252

[74] Wang T He C. TNF-α and IL-6: the link between immune and bone system. CDT (2020) 21:213–27. doi: 10.2174/1389450120666190821161259 31433756

[75] Maxwell LJ Zochling J Boonen A Singh JA Veras MM Tanjong Ghogomu E. TNF-alpha inhibitors for ankylosing spondylitis. Cochrane Database Syst Rev (2015) 2015(4):CD005468. doi: 10.1002/14651858.cd005468.pub2 PMC11200207 25887212

[76] Nigil Haroon N Sriganthan J Al Ghanim N Inman RD Cheung AM. Effect of TNF-alpha inhibitor treatment on bone mineral density in patients with ankylosing spondylitis: a systematic review and meta-analysis. Semin Arthritis Rheum (2014) 44:155–61. doi: 10.1016/j.semarthrit.2014.05.008 24909809

[77] Hakimian S Kheder J Arum S Cave DR Hyatt B. Re-evaluating osteoporosis and fracture risk in crohn’s disease patients in the era of TNF-alpha inhibitors. Scand J Gastroenterol (2017) 53:168–72. doi: 10.1080/00365521.2017.1416161 29235392

[78] Scheffler JM Grahnemo L Engdahl C Drevinge C Gustafsson KL Corciulo C. Interleukin 17A: a janus-faced regulator of osteoporosis. Sci Rep (2020) 10:5692. doi: 10.1038/s41598-020-62562-2 32231224 PMC7105470

[79] Toussirot É Wendling D. Antiinflammatory treatment with bisphosphonates in ankylosing spondylitis. Curr Opin Rheumatol (2007) 19:340–5. doi: 10.1097/bor.0b013e328133f57b 17551363

[80] Peris P Monegal A Guañabens N. Bisphosphonates in inflammatory rheumatic diseases. Bone (2021) 146:115887. doi: 10.1016/j.bone.2021.115887 33592328

[81] Eun I-S Park SH Goh TS Son SM Kim DS Lee JS. Effect of bisphosphonates on ankylosing spondylitis: a meta-analysis. J Clin Neurosci (2021) 92:153–8. doi: 10.1016/j.jocn.2021.08.016 34509243

[82] Jo S Han J Lee YL Yoon S Lee J Wang SE. Regulation of osteoblasts by alkaline phosphatase in ankylosing spondylitis. Int J Rheum Dis (2018) 22:252–61. doi: 10.1111/1756-185x.13419 30415492

[83] El Maghraoui A Tellal S Chaouir S Lebbar K Bezza A Nouijai A. Bone turnover markers, anterior pituitary and gonadal hormones, and bone mass evaluation using quantitative computed tomography in ankylosing spondylitis. Clin Rheumatol (2004) 24:346–51. doi: 10.1007/s10067-004-1039-8 15592691

[84] Watson CJ Tang WJ Rojas MF Fiedler IAK Morfin Montes de Oca E Cronrath AR. wnt16 regulates spine and muscle morphogenesis through parallel signals from notochord and dermomyotome. PloS Genet (2022) 18:e1010496. doi: 10.1371/journal.pgen.1010496 36346812 PMC9674140

[85] Li Z Wu X Leo PJ De Guzman E Akkoc N Breban M. Polygenic risk scores have high diagnostic capacity in ankylosing spondylitis. Ann Rheum Dis (2021) 80:1168–74. doi: 10.1136/annrheumdis-2020-219446 PMC8364478 34161253

[86] Montilla C Del Pino-Montes J Collantes-Estevez E Font P Zarco P Mulero J. Clinical features of late-onset ankylosing spondylitis: comparison with early-onset disease. J Rheumatol (2012) 39:1008–12. doi: 10.3899/jrheum.111082 22422491

[87] Mao SS Li D Syed YS Gao Y Luo Y Flores F. Thoracic quantitative computed tomography (QCT) can sensitively monitor bone mineral metabolism. Acad Radiol (2017) 24:1582–7. doi: 10.1016/j.acra.2017.06.013 28844601

[88] Li N Li X Xu L Sun W Cheng X Tian W. Comparison of QCT and DXA: osteoporosis detection rates in postmenopausal women. Int J Endocrinol (2013) 2013:1–5. doi: 10.1155/2013/895474 PMC3623474 23606843

