# Supplementary material for: Investigating the causal relationship between ankylosing spondylitis and osteoporosis in the European population: a bidirectional Mendelian randomization study
Source: Front Immunol. 2023 Jun 8;14:1163258. doi: 10.3389/fimmu.2023.1163258 (PMC10285397; doi:10.3389/fimmu.2023.1163258)
Supplement: Supplementary file 1 [file DataSheet_1.pdf]

Investigating the Causal Relationship between Ankylosing Spondylitis and Osteoporosis: A bidirectional Mendelian Randomization Study

The supplementary materials include:

1) Supplementary Tables 1-9
2) Supplementary Figures 1-8

1) Supplementary Tables 1-9

Supplementary Table 1. Details of the AS-associated SNP screening process in the Mendelian randomization analyses of AS on BMD at different sites and in different age groups.

SNP	FN-BMD	FA-BMD	TB-BMD	LS-BMD	Heel-BMD	0-15	15-30	30-45	45-60	60 or over
**rs11065898**	* rs73199895	* rs73199895		* rs10849951						
**rs11209026**	* rs140953244	* rs140953244		* rs140953244						
**rs1041926**										
**rs11190133**										
**rs1128905**										
**rs11624293**										
**rs130075**	/	/	/	/	/	/	/	/	/	/
**rs4129267**	* rs12133641			* rs12133641						
**rs41299637**	* rs905634			* rs905634						
**rs7191548**	* rs79533802	* rs79533802		* rs79533802						
**rs743479**	* rs762422			* rs762422						
**rs1250550**										
**rs12615545**										
**rs1801274**	×			×						
**rs1860545**										
**rs2517655**										
**rs2531875**										
**rs2596501**										
**rs27529**	×			×						
**rs2836883**										
**rs35164067**			* rs34953890							
**rs4672505**										
**rs4676410**										

rs6556416
rs6600247
rs9901869

/:missing required information for the MR tests;×: not aviable in outcome data and find no proxies;*: not aviable in outcome data but find proxies

Supplementary Table 2. Characteristics of the AS-associated genetic instrumental variables included in the MR study.

SNP	Chr	Pos	Gene	EA/OA	MAF	Beta	Se	*P*	R^2	F
**rs1041926**	6	28426296	OR2E1P	A/G	0.9851	-0.07483	0.011692	1.55E-10	0.003762451	85.52247627
rs11065898	12	1.12E+08	SH2B3	T/C	0.7903	0.026252	0.004806	4.71E-08	0.009103259	208.0371188
rs11190133	10	1.01E+08	NKX2-3	T/C	0.6978	-0.03387	0.004494	4.84E-14	0.009729913	222.4987679
rs11209026	1	67705958	IL23R	A/G	0.5984	-0.10358	0.009545	1.94E-27	0.004604872	104.7597268
rs1128905	9	1.39E+08	CARD9	C/T	0.4871	-0.02372	0.004095	6.95E-09	0.010668922	244.2031285
rs11624293	14	88488821	GPR65	C/T	0.0994	0.042868	0.006692	1.49E-10	0.006555447	149.4276696
rs1250550	10	81060317	ZMIZ1	A/C	0.7018	-0.02604	0.004305	1.46E-09	0.010153582	232.2864084
rs12615545	2	1.82E+08	UBE2E3	C/T	0.4414	0.025473	0.004173	1.03E-09	0.010471224	239.6300954
rs1801274#	1	1.61E+08	FCGR2A	G/A	0.5109	0.025318	0.004177	1.35E-09	0.010460954	239.3925806
rs1860545	12	6446777	UBE2E3	A/G	0.5775	-0.02747	0.004354	2.78E-10	0.010040155	229.6651848
rs2517655	6	30121048	TRIM10	T/C	0.2316	0.088286	0.004656	3.47E-80	0.009395073	214.7691968
rs2531875	17	26148167	NOS2	T/G	0.3887	-0.02732	0.004245	1.22E-10	0.010295678	235.5709892
rs2596501	6	31321211	HLA-B	T/C	0.5338	-0.15234	0.004194	1E-200	0.010418648	238.4142356
rs27529#	5	96126308	ERAP1	G/A	0.3499	-0.06204	0.004299	3.28E-47	0.010167751	232.6138776
rs2836883	21	40466744	PCP4	A/G	0.2575	-0.03968	0.004748	6.46E-17	0.009214254	210.5972831
rs35164067	19	10525181	TYK2	A/G	0.1680	-0.03108	0.004950	3.43E-10	0.008841154	201.9937918
rs4129267	1	1.54E+08	IL6R	T/C	0.6402	-0.03077	0.004226	3.32E-13	0.010340475	236.6066866
rs41299637	1	2.01E+08	C1orf106	G/T	0.2734	-0.03905	0.004910	1.81E-15	0.008912715	203.6434394
rs4672505	2	62560332	RP11-642D6.1	G/A	0.3748	-0.05978	0.004151	5.14E-47	0.010525338	240.8816295
rs4676410	2	2.42E+08	GPR35	A/G	0.8012	0.028101	0.004902	9.9E-09	0.008927058	203.974111
rs6556416	5	1.59E+08	AC008697.1	C/A	0.3042	0.025215	0.004600	4.22E-08	0.009507445	217.3626556
rs6600247	1	25305114	RUNX3	C/T	0.5457	0.032833	0.004151	2.58E-15	0.010525764	240.8914948
rs7191548	16	28614734	SULT1A1	C/T	0.6650	0.024985	0.004285	5.51E-09	0.010199834	233.3554313

rs743479	21	45611950	CH507-9B2.8	T/C	0.6133	-0.02342	0.004176	2.03E-08	0.010463582	239.4533487
rs9901869	17	45575206	MRPL45P2	A/G	0.5030	0.031904	0.004089	6.04E-15	0.010684567	244.5650853

#: The variants rs1801274 and rs27529 were not included as instrumental variables in the MR analysis for AS and FN-BMD, as well as AS and LS-BMD, due to their absence in the FN-BMD and LS-BMD dataset.

Supplementary Table 3. Likely causal associations of the instrumental SNPs with Ankylosing spondylitis.

SNP	Chromosome	Likely causal gene at locus	Functional link with AS	References
**rs11065898**	12	*SH2B3*	SH2B3 is involved in CD8+, CD4+ T cells differentiation and probably in AS susceptibility.[1]	Soleimanifar N, Assadiasl S (2022) Overview on Signal Transduction. Ankylosing Spondylitis-Axial Spondyloarthritis. p. 116–126
**rs11209026**	1	*IL23R*	IL-23 and IL-23 receptors are the essential parts in Th17 regulation.[1] IL23R is associated with increased Th1-cell differentiation.[2]	PMID: 26916345
**rs1128905**	9	*CARD9*	CARD9 induce the canonical NF-κB pathway and also p38 mitogen-activated protein kinase (MAPK) and c-Jun N-terminal kinase (JNK) pathways for pro-inflammatory responses[1]Deletion of CARD9 in DCs alone reversed the development of autoimmunity and colitis in DC-specific Lyn-deficient mice.[3]	PMID: 31594855
**rs11624293**	14	*GPR65*	T cell death-associated gene 8 (TRAG8) is a GPR65 mouse homolog, leading the inhibition of pro-	Soleimanifar N, Assadiasl S (2022) Overview on Signal Transduction. Ankylosing Spondylitis-Axial Spondyloarthritis. p. 116–126

			inflammatory cytokines and also inducing thymocyte apoptosis.[1]	
**rs1250550**	10	*ZMIZ1*	ZMIZ1 involved in CD8+, CD4+ T cells differentiation and probably in AS susceptibility.[1]	Soleimanifar N, Assadiasl S (2022) Overview on Signal Transduction. Ankylosing Spondylitis-Axial Spondyloarthritis. p. 116–126
**rs1860545**	12	*UBE2E3*	UBE2L3 variants are involved in the regulation of NF-κB and it has been associated with a number of other inflammatory disorders.[1]	Soleimanifar N, Assadiasl S (2022) Overview on Signal Transduction. Ankylosing Spondylitis-Axial Spondyloarthritis. p. 116–126
**rs27529**	5	*ERAP1*	ERAP1 is involved in trimming peptides to optimal length for binding to HLA class 1 molecules, thereby affecting the stability and processing of HLA-B27.[1][4]	PMID: 23452840
**rs35164067**	19	*TYK2*	TYK2 inhibition reduces type 3 immunity and modifies disease progression in murine spondyloarthritis.[5]	PMID: 32149730
**rs4129267**	1	*IL6R*	Interleukin-6 is effective in inducing inflammation in both immune defense and autoimmune conditions.[1]	Soleimanifar N, Assadiasl S (2022) Overview on Signal Transduction. Ankylosing Spondylitis-Axial Spondyloarthritis. p. 116–126
**rs41299637**	1	*GPR25 - KIF21B*	GPR25 - KIF21B exerting a very consequential role in the activation of immune cells and the formation of immune-mediated inflammation.[1]	Soleimanifar N, Assadiasl S (2022) Overview on Signal Transduction. Ankylosing Spondylitis-Axial Spondyloarthritis. p. 116–126
**rs4676410**	2	*GPR35*	Transmission of the indoleamine 2, 3-dioxygenase) IDO (signal, which is the major mediator in the immune system and causes tolerance in dendritic cells, is done by GPR35.[1]	Soleimanifar N, Assadiasl S (2022) Overview on Signal Transduction. Ankylosing Spondylitis-Axial Spondyloarthritis. p. 116–126
**rs6600247**	1	*RUNX3*	RUNX3 is a key gene in CD8 lymphocyte differentiation.[1] RUNX3	PMID: 30687330

			is involved in the TGFβ signaling pathway.[6]	
**rs9901869**	17	*NPEPPS-TBKBP1-TBX21*	TBKBP1 is involved in TNF signaling pathway in AS pathogenesis.[1]	Soleimanifar N, Assadiasl S (2022) Overview on Signal Transduction. Ankylosing Spondylitis-Axial Spondyloarthritis. p. 116–126

1. Soleimanifar N, Assadiasl S (2022) Overview on Signal Transduction. Ankylosing Spondylitis-Axial Spondyloarthritis. p. 116–126
2. Roberts AR, Vecellio M, Chen L, et al. An ankylosing spondylitis-associated genetic variant in the IL23R-IL12RB2 intergenic region modulates enhancer activity and is associated with increased Th1-cell differentiation. Ann Rheum Dis. 2016;75(12):2150-2156. doi:10.1136/annrheumdis-2015-208640
3. Ma J, Abram CL, Hu Y, Lowell CA. CARD9 mediates dendritic cell-induced development of Lyn deficiency-associated autoimmune and inflammatory diseases. Sci Signal. 2019;12(602):eaao3829. Published 2019 Oct 8. doi:10.1126/scisignal.aao3829
4. Keidel S, Chen L, Pointon J, Wordsworth P. ERAP1 and ankylosing spondylitis. Curr Opin Immunol. 2013;25(1):97-102. doi:10.1016/j.coi.2012.11.002

5. Gracey E, Hromadová D, Lim M, et al. TYK2 inhibition reduces type 3 immunity and modifies disease progression in murine spondyloarthritis. J Clin Invest. 2020;130(4):1863-1878. doi:10.1172/JCI126567
6. Vecellio M, Cohen CJ, Roberts AR, Wordsworth PB, Kenna TJ. RUNX3 and T-Bet in Immunopathogenesis of Ankylosing Spondylitis-Novel Targets for Therapy?. Front Immunol. 2019;9:3132. Published 2019 Jan 10. doi:10.3389/fimmu.2018.03132

Supplementary Table 4. The reported traits of selected instrumental variables of AS searched in phenoscanner.

SNP	Gene	Trait(s)
**rs1041926**	OR2E1P	Rheumatoid arthritis
**rs11065898**	SH2B3	White blood cell count; Self-reported hypothyroidism or myxoedema; Low density lipoprotein; Coronary artery disease
**rs11190133**	NKX2-3	Inflammatory bowel disease; Plateletcrit; Monocyte count
**rs11209026**	IL23R	Inflammatory bowel disease; Irritible bowel syndrome; Blood protein levels
**rs1128905**	CARD9	Inflammatory bowel disease; Fasting glucose
**rs11624293**	GPR65	NA
**rs1250550**	ZMIZ1	Inflammatory bowel disease; Multiple sclerosis
**rs12615545**	UBE2E3	Monocyte percentage of white cells
**rs1801274**	FCGR2A	Inflammatory bowel disease; Systemic lupus erythematosus; Total cholesterol
**rs1860545**	UBE2E3	Primary biliary cirrhosis; Monocyte count; Alopecia areata
**rs2517655**	TRIM10	Rheumatoid arthritis ; White blood cell count ; Self-reported malabsorption or coeliac disease
**rs2531875**	NOS2	NA
**rs2596501**	HLA-B	Rheumatoid arthritis; Self-reported psoriasis; Weight; Type 1 diabetes; Self-reported hypothyroidism or myxoedema
**rs27529**	ERAP1	NA
**rs2836883**	Intergenic	Inflammatory bowel disease; Primary sclerosing cholangitis; Neutrophil count
**rs35164067**	TYK2	Inflammatory bowel disease; Lymphocyte count; Self-reported psoriasis
**rs4129267**	IL6R	Rheumatoid arthritis; C-reactive protein; Allergic disease
**rs41299637**	GPR25-*KIF21B*	Inflammatory bowel disease; Lymphocyte percentage of white cells
**rs4672505**	intergenic; RP11-642D6.1	NA
**rs4676410**	GPR35	Inflammatory bowel disease; Monocyte count
**rs6556416**	AC008697.1	Inflammatory bowel disease; Self-reported psoriasis ; Mouth or teeth dental problems: mouth ulcers
**rs6600247**	RUNX3	NA
**rs7191548**	SULT1A1	Crohns disease; Whole body fat mass; Alcohol intake frequency
**rs743479**	CH507-9B2.8	Inflammatory bowel disease
**rs9901869**	NPEPPS-TBKBP1-TBX21	Multiple sclerosis; Height

Supplementary Table 5. Characteristics of the BMD-associated genetic instrumental variables included in the MR study.

SNP	Chr	Pos	Gene	EA/OA	MAF	Beta	Se	*P*	R²	F
**Instrumental variables of TB-BMD**										
rs2252865	1	8422676	RERE	C/T	0.6759	0.0328	0.006	4.72E-08	0.002952	166.6607
rs10493013	1	22703035	RP11-415K20.2	C/T	0.1811	0.1013	0.0074	4.07E-43	0.002395	135.1303
rs11904127	2	85484818	TCF7L1	A/G	0.5512	-0.0324	0.0057	1.18E-08	0.003107	175.4324
rs13204965	6	127167072	RP11-394G3.2	C/A	0.229	-0.0619	0.007	1.02E-18	0.002532	142.8521
rs3801387	7	120974765	WNT16	G/A	0.2721	0.1347	0.0063	1.15E-100	0.002812	158.7245
rs1548607	7	50901491	AC004920.3	G/A	0.313	-0.0363	0.0066	4.18E-08	0.002685	151.5098
rs61884327	11	46766890	CKAP5	C/T	0.0978	0.0801	0.0099	4.63E-16	0.001791	101.0065
rs11228240	11	68218290	LRP5	T/C	0.2574	-0.083	0.0067	1.72E-35	0.002645	149.2484
**Instrumental variables of LS-BMD**										
rs7524102	1	22698447	RP11-415K20.2	G/A	0.198329	0.089822	0.011494	2.41E-14	0.003044	86.99581
rs1023940	6	151932778	CCDC170	G/A	0.534845	-0.06454	0.00876	6.47E-13	0.00399	114.1472
rs1357651	7	38097862	SFRP4	G/T	0.645187	0.068117	0.009151	3.75E-13	0.00382	109.27
rs7807953	7	121000718	FAM3C	T/C	0.263325	0.075102	0.009698	4.11E-14	0.003605	103.1068
rs2291467	11	68216756	LRP5	T/C	0.228162	-0.07742	0.010148	9.64E-14	0.003446	98.53467
**Instrumental variables of Heel-BMD**										
rs6684375	1	22706434	RP11-415K20.2	T/C	0.176265	0.062122	0.00334	3.40E-77	0.001126	299.3613
rs3765971	1	8445360	RERE	T/C	0.657495	0.028468	0.002684	2.70E-26	0.001401	372.6324
rs4912084	1	19721548	CAPZB	G/A	0.622386	-0.01781	0.00263	1.30E-11	0.00143	380.2759
rs13022041	2	25400559	POMC	T/G	0.325615	0.017788	0.00273	7.20E-11	0.001377	366.3405
rs12636449	3	50180613	SEMA3F-AS1	G/A	0.085448	0.0436	0.004576	1.60E-21	0.000822	218.5246
rs1991431	3	141133450	ZBTB38	A/G	0.441046	-0.01529	0.002577	2.90E-09	0.001459	388.1024
rs6532480	4	95277038	MANBA	C/T	0.43972	-0.01558	0.002585	1.70E-09	0.001454	386.9056
rs7699480	4	145607846	HHIP	T/C	0.56138	-0.01958	0.002573	2.70E-14	0.001461	388.6515
rs6882422	5	135430668	CTB-1I21.1	A/G	0.116759	-0.03137	0.004006	4.90E-15	0.000939	249.6193
rs2069443	7	150755173	CDK5	G/T	0.255369	-0.01774	0.002914	1.10E-09	0.00129	343.1342

rs11228240	11	68218290	LRP5	T/C	0.276559	-0.04059	0.002858	8.70E-46	0.001316	349.8961
rs2553772	11	35085453	PDHX	G/T	0.540261	0.030946	0.002566	1.70E-33	0.001465	389.7087
rs13379337	14	35224430	BAZ1A	A/C	0.488789	0.019901	0.002573	1.00E-14	0.001461	388.6333
rs12915039	15	67434348	SMAD3	C/A	0.240277	0.019891	0.003033	5.50047E-11	0.00124	329.6552
rs17601876	15	51553909	CYP19A1	G/A	0.478533	0.030301	0.002567	3.69999E-32	0.001465	389.5903
rs4807630	19	1170445	SBNO2	T/C	0.310033	-0.03333	0.002789	6.4998E-33	0.001348	358.514
rs56257969	19	57797752	ZNF460	G/A	0.259313	-0.016	0.002928	4.60002E-08	0.001284	341.538

Supplementary Table 6. The reported traits of selected genetic instrumental variables of BMD searched in phenoscanner.

SNP	Gene	Trait(s)
**Instrumental variables of TB-BMD**		
rs2252865	RERE	Heel bone mineral density; Systolic blood pressure
rs10493013	RP11-415K20.2	Inflammatory bowel disease; Ulcerative colitis; Heel bone mineral density;Immature fraction of reticulocytes
rs11904127	TCF7L1	NA
rs13204965	RP11-394G3.2	Height; Whole body fat-free mass; Self-reported hypertension
rs3801387	WNT16	Heel bone mineral density; Fracture of forearm
rs1548607	AC004920.3	Heel bone mineral density; Self-reported osteoporosis
rs61884327	CKAP5	Whole body fat-free mass
rs11228240	LRP5	Heel bone mineral density; Trunk fat-free mass
**Instrumental variables of LS-BMD**		
rs7524102	RP11-415K20.2	Inflammatory bowel disease; Ulcerative colitis; Red cell distribution width; Heel bone mineral density
rs1023940	CCDC170	Heel bone mineral density
rs1357651	SFRP4	Heel bone mineral density; Fibroblastic disorders
rs7807953	FAM3C	Heel bone mineral density; Forearm bone mineral density
rs2291467	LRP5	Heel bone mineral density; Basal metabolic rate
**Instrumental variables of Heel-BMD**		
rs56257969	ZNF460	NA
rs1991431	ZBTB38	Allergic disease;Basal metabolic rate
rs13022041	POMC	Trunk predicted mass
rs17601876	CYP19A1	Heel bone mineral density
rs6684375	RP11-415K20.2	Inflammatory bowel disease; Crohn's disease;Red cell distribution width
rs12636449	SEMA3F-AS1	Body mass index; Pulse rate
rs2553772	PDHX	Whole body water mass;Birth weight
rs12915039	SMAD3	NA
rs7699480	HHIP	Basal metabolic rate ; Basal metabolic rate
rs2069443	CDK5	Basal metabolic rate; Whole body water mass

rs4912084	CAPZB	NA
rs11228240	LRP5	Whole body water mass;Trunk fat-free mass
rs3765971	RERE	Allergic disease; Schizophrenia;Hayfever, allergic rhinitis or eczema
rs6882422	CTB-1I21.1	NA
rs6532480	MANBA	NA
rs13379337	BAZ1A	NA
rs4807630	SBNO2	Immature fraction of reticulocytes;Reticulocyte count

Supplementary Table 7. Statistical power for the Mendelian randomization analyses of AS on BMD at different sites and in different age groups.

Exposures	Outcomes	Sample size of outcome	Type-I error rate	number of IVs	variance	β_{OLS}	range of β_{yx} (OR) when the statistical power is more than 80%
**AS**	TB-BMD	56284	0.05	25	0.23397012	-0.0166132	⩾0.0243(1.025) or ⩽-0.0243(0.976)
**AS**	FN-BMD	32735	0.05	23	0.21334141	-0.00070354	⩾0.0334(1.034) or ⩽-0.0334(0.967)
**AS**	LS-BMD	28498	0.05	23	0.21334141	0.012647147	⩾0.0358(1.036) or ⩽-0.0358(0.965)
**AS**	FA-BMD	8143	0.05	25	0.23397012	-0.05011947	⩾0.0642(1.066) or ⩽-0.0642(0.938)
**AS**	Heel-BMD	265627	0.05	25	0.23397012	0.007193165	⩾0.0112(1.011) or ⩽-0.0112(0.989)
**AS**	TB-BMD(0-15)	11807	0.05	25	0.23397012	-0.03970847	⩾0.0532(1.055) or ⩽-0.0532(0.948)
**AS**	TB-BMD(15-30)	4180	0.05	25	0.23397012	0.10179852	⩾0.0886(1.093) or ⩽-0.0886(0.915)
**AS**	TB-BMD(30-45)	10062	0.05	25	0.23397012	0.07855892	⩾0.0573(1.059) or ⩽-0.0573(0.944)
**AS**	TB-BMD(45-60)	18805	0.05	25	0.23397012	-0.03045903	⩾0.0421(1.043) or ⩽-0.0421(0.959)
**AS**	TB-BMD(60 or more)	22504	0.05	25	0.23397012	-0.06118784	⩾0.0386(1.039) or ⩽-0.0386(0.962)

β_{OLS}, the causal association by MR analysis in IVW between X and Y (AS and TB-BMD for instance)

βyx, hypothetical values of the unknown true causal association between X and Y (AS and TB-BMD for instance)

Supplementary Table 8. Statistical power for the Mendelian randomization analyses of BMD on AS.

Exposure	Outcome	variance	Sample size of outcome	Proportion of cases	Type-I error rate	Odds ratio of outcome	Statistical power
TB-BMD	AS	0.02091989	22647	0.400450391	0.05	0.8	65 %
LS-BMD	AS	0.01790447	22647	0.400450391	0.05	0.8	58%
Heel-BMD	AS	0.02233646	22647	0.400450391	0.05	0.8	67%

Odds ratio of outcome, hypothetical values of the unknown true causal association between X and Y (TB-BMD and AS for instance)

Supplementary Table 9. Overlapping genetic loci, genes, and functional pathways in ankylosing spondylitis and bone mineral density changes.

SNP	Gene	signal pathway	function of genes
**rs1041926**	OR2E1P	unknown	the OR2E1P gene is a pseudogene and belongs to the Olfactory Receptor Family 2 Subfamily E Member 1 Pseudogene. Olfactory receptors interact with odorant molecules in the nose to initiate a neuronal response that triggers the perception of a smell. The olfactory receptor proteins are members of a large family of G-protein-coupled receptors (GPCR) arising from single coding-exon genes .
**rs11065898**	SH2B3	Insulin signaling pathway, JAK-STAT signaling pathway, T-cell receptor signaling pathway, etc.	The SH2B3 gene encodes the SH2B3 protein, also known as lymphocyte adapter protein (LNK). It is ubiquitously expressed in many tissues and cell types. LNK functions as a regulator in signaling pathways relating to hematopoiesis, inflammation, and cell migration.
**rs11190133**	NKX2-3	unknown	Potential role in cell differentiation
**rs11209026**	IL23R	IL-23 signaling pathway, Th17 cell differentiation, etc.	Promotes inflammation and helps coordinate the immune system's response to foreign invaders (e.g. bacteria and viruses)
**rs1128905**	CARD9	NF-kappa B signaling pathway, Fc epsilon RI signaling pathway, Toll-like receptor signaling pathway, etc.	The CARD9 gene provides instructions to make an immune system protein involved in the body's defence against fungal infection
**rs11624293**	GPR65	unknown	The GPR65 gene encodes a protein that is a G protein-coupled receptor that senses pH
**rs1250550**	ZMIZ1	unknown	The protein encoded by the ZMIZ1 gene is a member of the PIAS (Protein Inhibitor Activated STAT) family. This protein regulates the activity of several transcription factors, including the androgen receptor, Smad3/4 and p53
**rs12615545**	UBE2E3	Ubiquitin mediated proteolysis, Wnt signaling pathway, etc.	The protein encoded by the UBE2E3 gene is a ubiquitin-binding enzyme E2 E3. Modification of the protein with ubiquitin is an important cellular mechanism for targeting abnormal or short-lived proteins for degradation
**rs1801274**	FCGR2A	Fc gamma R-mediated phagocytosis, Chemokine signaling pathway, etc.	The FCGR2A gene encodes a protein that is a cell surface receptor, once known as CD32, which is located at the 1q23 locus and whose gene product is expressed on a variety of cell types and mediates a variety of cell type-specific functions, including phagocytosis, release of inflammatory mediators and clearance of immune complexes 1
**rs1860545**	UBE2E3	Ubiquitin mediated proteolysis, Wnt signaling pathway, etc.	The protein encoded by the UBE2E3 gene is a ubiquitin-binding enzyme E2 E3.

			Modification of the protein with ubiquitin is an important cellular mechanism for targeting abnormal or short-lived proteins for degradation
**rs2517655**	TRIM10	TNF-alpha/NF-kappa B signaling pathway, Influenza A, etc.	The protein encoded by the TRIM10 gene is an E3 ligase that plays an important role in the differentiation and survival of terminal erythrocytes. It may bind directly to PTEN and promote its ubiquitination, leading to its proteasomal degradation and the activation of hypertrophic signalling
**rs2531875**	NOS2	NO signaling pathway, VEGF signaling pathway, TNF signaling pathway, etc.	NOS2 (nitric oxide synthase 2) is a gene that encodes for an enzyme called inducible nitric oxide synthase (iNOS), which catalyzes the production of nitric oxide (NO) from L-arginine. iNOS is primarily expressed in immune cells and is involved in various physiological and pathological processes, including inflammation, immune response, and host defense against pathogens.
**rs2596501**	HLA-B	Antigen processing and presentation, Immune response, etc.	HLA-B (human leukocyte antigen B) is a gene that encodes for a protein called HLA-B, which is a major histocompatibility complex (MHC) class I molecule. HLA-B molecules present antigenic peptides to cytotoxic T cells, which play a critical role in immune response against pathogens and tumors.
**rs27529**	ERAP1	Antigen processing and presentation, Protein processing in endoplasmic reticulum, etc.	ERAP1 (endoplasmic reticulum aminopeptidase 1) is a gene that encodes for an enzyme called ERAP1, which is involved in the processing and presentation of antigenic peptides by MHC class I molecules. ERAP1 trims the antigenic peptides to the appropriate length for presentation by MHC class I molecules.
**rs2836883**	Intergenic	unknown	NA
**rs35164067**	TYK2	JAK-STAT signaling pathway, Cytokine-cytokine receptor interaction, etc.	TYK2 (tyrosine kinase 2) is a gene that encodes for a protein called TYK2, which is a member of the Janus kinase (JAK) family. TYK2 is involved in various signaling pathways, including those that are activated by cytokines, and plays a role in immune response and inflammation.
**rs4129267**	IL6R	IL-6 signaling pathway, JAK-STAT signaling pathway, etc.	L6R (interleukin 6 receptor) is a gene that encodes for a protein called IL6R, which is a receptor for the cytokine interleukin 6 (IL-6). IL-6 is involved in various physiological and pathological processes, including inflammation and immune response. IL6R plays a critical role in the activation of downstream signaling pathways in response to IL-6.
**rs41299637**	GPR25-*KIF21B*	unknown	GPR25-KIF21B is a region on chromosome 1 that contains two genes, GPR25 and KIF21B. GPR25 encodes for a G protein-coupled receptor that is involved in various physiological and pathological processes, including inflammation and

			neurodevelopment. KIF21B encodes for a kinesin protein that is involved in intracellular transport.
**rs4672505**	intergenic; RP11-642D6.1	unknown	NA
**rs4676410**	GPR35	unknown	The protein encoded by the GPR35 gene is a G protein-coupled receptor that plays a role as a risk gene in intestinal diseases. It has been shown to be activated by the metabolite tryptophan-derived KYNA, the chemokine CXCL17 and the phospholipid derivative LPA species. Since its identification as a risk gene for IBD, there have been studies on GPR35 in intestinal disease
**rs6556416**	AC008697.1	unknown	NA
**rs6600247**	RUNX3	TGF-beta signaling pathway, Notch signaling pathway, etc.	RUNX3 (runt-related transcription factor 3) encodes for a transcription factor that plays a crucial role in the development and differentiation of various cell types, including T cells, dendritic cells, and epithelial cells. RUNX3 regulates the expression of genes involved in cell proliferation, differentiation, and apoptosis, and is involved in various physiological and pathological processes, including immune response, inflammation, and cancer.
**rs7191548**	SULT1A1	Metabolism of xenobiotics by cytochrome P450, Drug metabolism - cytochrome P450, etc.	SULT1A1 (sulfotransferase 1A1) encodes for an enzyme that is involved in the detoxification of xenobiotics and endogenous compounds by catalyzing the sulfation of various substrates, including hormones, drugs, and environmental toxins.
**rs743479**	CH507-9B2.8	unknown	NA
**rs9901869**	NPEPPS-TBKBP1-TBX21	RIG-I-like receptor signaling pathway, TLR signaling pathway, etc.	NPEPPS (aminopeptidase puromycin sensitive), TBKBP1 (TANK binding kinase 1 binding protein 1), and TBX21 (T-box transcription factor 21) are three genes that are located in close proximity to each other on chromosome 17. NPEPPS encodes for an enzyme that is involved in the degradation of peptide hormones and neurotransmitters. TBKBP1 encodes for a protein that regulates the activity of the protein kinase TBK1, which is involved in various signaling pathways, including those that are activated by viral infection. TBX21 encodes for a transcription factor that plays a critical role in the differentiation and function of T cells. Genetic variations in these genes have been associated with susceptibility to various diseases, including autoimmune disorders and infectious diseases.
**rs2252865**	RERE	Notch signaling pathway, Wnt signaling pathway, etc.	RERE (Arginine-Glutamic Acid Dipeptide Repeats) encodes for a transcriptional regulator that is involved in the regulation of gene expression during embryonic

			development, and also plays a role in cell signaling and differentiation in adults.
**rs10493013**	RP11-415K20.2	unknown	NA
**rs11904127**	TCF7L1	Wnt signaling pathway, Notch signaling pathway, etc.	TCF7L1 (Transcription factor 7-like 1) encodes for a transcription factor that is involved in the regulation of gene expression during embryonic development, particularly in the formation of the gut and other organs. TCF7L1 also plays a role in the development of immune cells and the maintenance of the intestinal epithelium.
**rs13204965**	RP11-394G3.2	unknown	NA
**rs3801387**	WNT16	Wnt signaling pathway, Hippo signaling pathway, etc.	WNT16 (Wingless-type MMTV integration site family, member 16) encodes for a protein that is involved in the Wnt signaling pathway, which plays a critical role in the regulation of cell growth, differentiation, and apoptosis.
**rs1548607**	AC004920.3	unknown	NA
**rs61884327**	CKAP5	Cell cycle, DNA replication, etc.	CKAP5 (Cytoskeleton-associated protein 5) encodes for a protein that is involved in the organization and stability of microtubules, which are essential components of the cytoskeleton
**rs11228240**	LRP5	Wnt signaling pathway, Notch signaling pathway, etc.	LRP5 (Low-density lipoprotein receptor-related protein 5) encodes for a protein that is involved in the regulation of bone density and the Wnt signaling pathway.
**rs7524102**	RP11-415K20.2	unknown	NA
**rs1023940**	CCDC170	unknown	CCDC170 (Coiled-coil domain-containing protein 170) encodes for a protein that is involved in the regulation of microtubule dynamics and cell division.
**rs1357651**	SFRP4	Wnt signaling pathway, etc.	SFRP4 (Secreted frizzled-related protein 4) encodes for a protein that is involved in the Wnt signaling pathway, which plays a critical role in the regulation of cell growth, differentiation, and apoptosis.
**rs7807953**	FAM3C	unknown	FAM3C (Family with sequence similarity 3 member C) encodes for a protein that is involved in the regulation of insulin secretion and glucose metabolism.
**rs2291467**	LRP5	Wnt signaling pathway, Notch signaling pathway, etc.	LRP5 (Low-density lipoprotein receptor-related protein 5) encodes for a protein that is involved in the Wnt signaling pathway, which plays a crucial role in cell growth, differentiation, and development. LRP5 is also important for bone formation and remodeling, as it regulates the activity of osteoblasts and osteoclasts.
**rs6684375**	RP11-415K20.2	unknown	NA
**rs3765971**	RERE	Notch signaling pathway, Wnt signaling pathway, etc.	RERE (Arginine-Glutamic Acid Dipeptide Repeats) encodes for a transcriptional regulator that is involved in the regulation of gene expression during embryonic development, and also plays a role in cell signaling and differentiation in adults.

**rs4912084**	CAPZB	Hippo signaling pathway, Regulation of actin cytoskeleton, etc.	CAPZB (Capping protein, muscle Z-line, beta subunit) encodes for a protein that is involved in the regulation of actin filament dynamics and cytoskeleton organization.
**rs13022041**	POMC	Insulin signaling pathway, Adipocytokine signaling pathway, etc.	POMC (Pro-opiomelanocortin) encodes for a protein precursor that is processed into several biologically active peptides, including melanocyte-stimulating hormone (MSH) and beta-endorphin. POMC plays a critical role in the regulation of appetite, stress response, and pain sensation.
**rs12636449**	SEMA3F-AS1	actin cytoskeleton pathway, MAPK signaling pathway, Wnt signaling pathway	NA
**rs1991431**	ZBTB38	Cell cycle pathway, DNA replication pathway, Notch signaling pathway	ZBTB38 (Zinc finger and BTB domain-containing protein 38) encodes for a transcription factor that is involved in the regulation of gene expression and cell differentiation.
**rs6532480**	MANBA	Lysosome pathway, Glycosaminoglycan degradation pathway	MANBA (Mannosidase beta A) encodes for an enzyme that is involved in the degradation of glycoproteins and glycolipids.
**rs7699480**	HHIP	Hedgehog signaling pathway, TGF-beta signaling pathway, Wnt signaling pathway	HHIP (Hedgehog-interacting protein) encodes for a protein that is involved in the regulation of the Hedgehog signaling pathway, which plays a crucial role in embryonic development and tissue regeneration.
**rs6882422**	CTB-1I21.1	unknown	NA
**rs2069443**	CDK5	Cell cycle pathway, Axon guidance pathway, MAPK signaling pathway	CDK5 (Cyclin-dependent kinase 5) encodes for a protein kinase that is involved in various cellular processes, including neuronal development and function, and cell cycle regulation.
**rs2553772**	PDHX	Pyruvate metabolism pathway, Citrate cycle pathway	PDHX (Pyruvate dehydrogenase complex component X) encodes for a protein that is involved in the regulation of the pyruvate dehydrogenase complex, which plays a crucial role in cellular energy metabolism.
**rs13379337**	BAZ1A	Chromatin remodeling pathway, DNA repair pathway, Notch signaling pathway	BAZ1A (Bromodomain adjacent to zinc finger domain protein 1A) encodes for a protein that is involved in chromatin remodeling and gene expression regulation.
**rs12915039**	SMAD3	TGF-beta signaling pathway, MAPK signaling pathway, Wnt signaling pathway	SMAD3 (Mothers against decapentaplegic homolog 3) encodes for a protein that is involved in the TGF-beta signaling pathway, which plays a crucial role in cell growth, differentiation, and development.
**rs17601876**	CYP19A1	Steroid hormone biosynthesis pathway, Ovarian steroidogenesis	CYP19A1 (Cytochrome P450 family 19 subfamily A member 1) encodes for an enzyme that is involved in the synthesis of estrogen from androgen precursors.
**rs4807630**	SBNO2	Notch signaling, TGF-beta signaling, and Wnt signaling.	SBNO2 (Strawberry notch homolog 2) encodes for a transcription factor that is involved in the regulation of gene expression and cell differentiation.

**rs56257969**	ZNF460	unknown	NA

2) Supplementary Figures 1-8

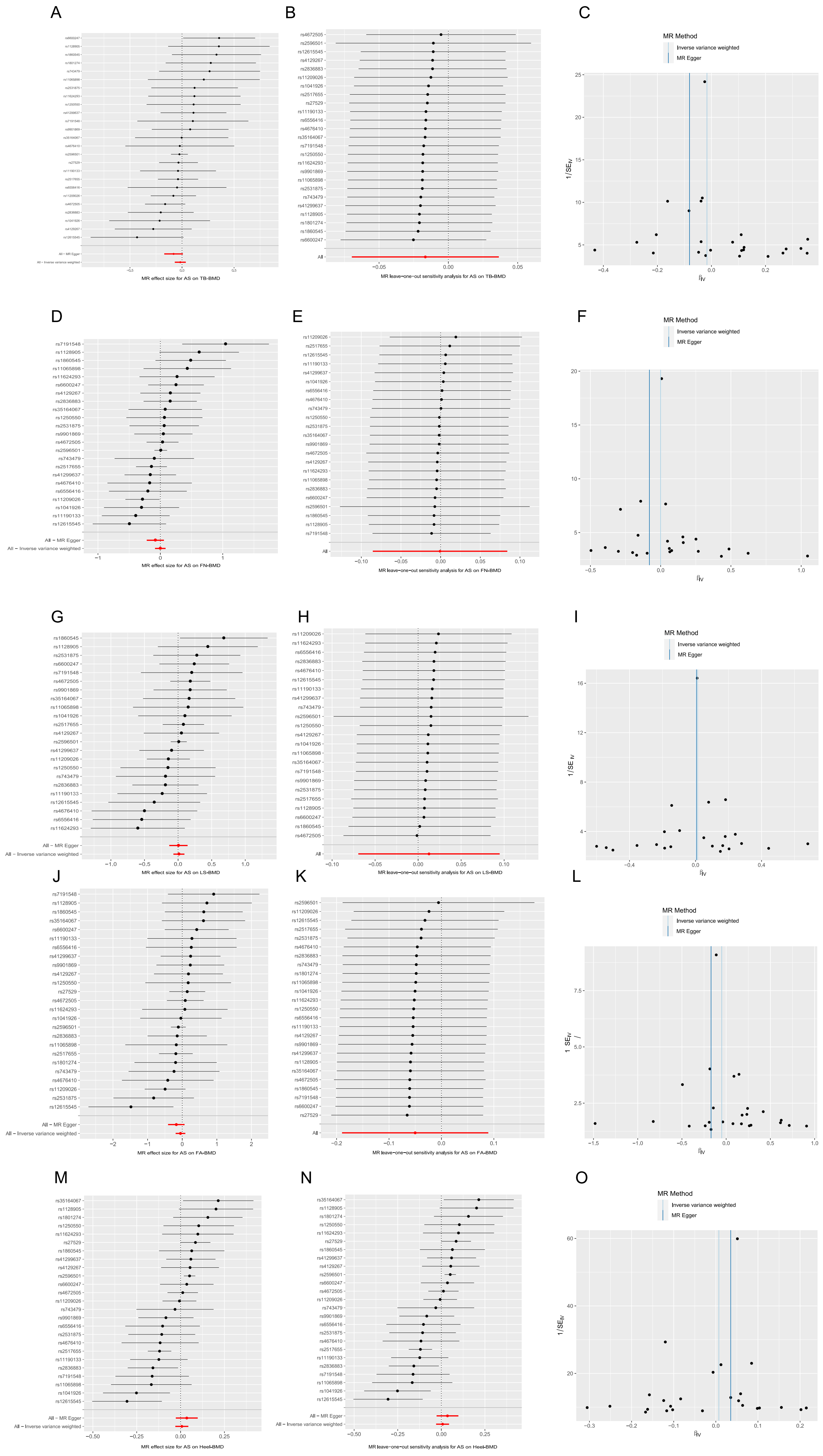
A
MR effect size for AS on TB-BMD
B
MR leave-one-out sensitivity analysis for AS on TB-BMD
C
MR Method
Inverse variance weighted
MR Egger
D
MR effect size for AS on FN-BMD
E
MR leave-one-out sensitivity analysis for AS on FN-BMD
F
MR Method
Inverse variance weighted
MR Egger
G
MR effect size for AS on LS-BMD
H
MR leave-one-out sensitivity analysis for AS on LS-BMD
I
MR Method
Inverse variance weighted
MR Egger
J
MR effect size for AS on FA-BMD
K
MR leave-one-out sensitivity analysis for AS on FA-BMD
L
MR Method
Inverse variance weighted
MR Egger
M
MR effect size for AS on Heel-BMD
N
MR leave-one-out sensitivity analysis for AS on Heel-BMD
O
MR Method
Inverse variance weighted
MR Egger
All − MR Egger
All − Inverse variance weighted

Supplementary Figure 1. MR sensitivity analysis of AS on BMD by site.

Forest plot(A), leave-one-out sensitivity analysis(B) and funnel plot (C) of the effect of AS on TB-BMD; forest plot(D), leave-one-out sensitivity analysis(E) and funnel plot (F) of the effect of AS on FN-BMD; forest plot(G), leave-one-out sensitivity analysis(H) and funnel plot (I) of the effect of AS on LS-BMD; forest plot(J), leave-one-out sensitivity analysis(K) and funnel plot (L) of the effect of AS on FA-BMD; forest plot(M), leave-one-out sensitivity analysis(N) and funnel plot (O) of the effect of AS on Heel-BMD. AS: Ankylosing spondylitis; TB-BMD: total body bone mineral density; FN-BMD: femoral neck bone mineral density; LS-BMD: lumbar spine bone mineral density; FA-BMD: forearm bone mineral density; Heel-BMD: heel bone mineral density.

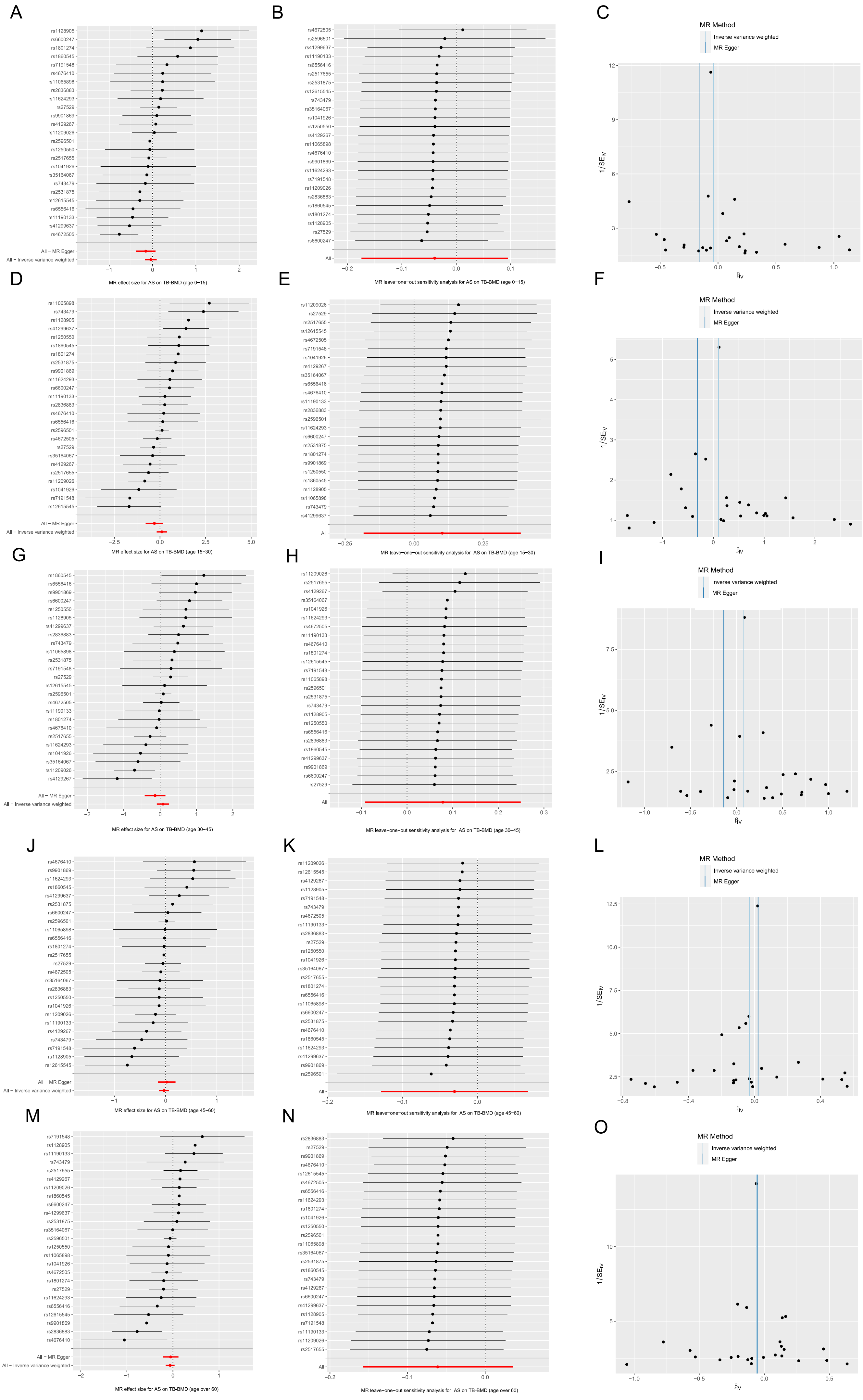
A
B
C
D
E
F
G
H
I
J
K
L
M
N
O
MR effect size for AS on TB-BMD (age 0–15)
MR leave-one-out sensitivity analysis for AS on TB-BMD (age 0–15)
MR effect size for AS on TB-BMD (age 15–30)
MR leave-one-out sensitivity analysis for AS on TB-BMD (age 15–30)
MR effect size for AS on TB-BMD (age 30–45)
MR leave-one-out sensitivity analysis for AS on TB-BMD (age 30–45)
MR effect size for AS on TB-BMD (age 45–60)
MR leave-one-out sensitivity analysis for AS on TB-BMD (age 45–60)
MR effect size for AS on TB-BMD (age over 60)
MR leave-one-out sensitivity analysis for AS on TB-BMD (age over 60)
All – MR Egger
All – Inverse variance weighted
MR Method
Inverse variance weighted
MR Egger
$1/SE_{IV}$
β_{IV}

Supplementary Figure 2. MR sensitivity analysis of AS on BMD at different ages.

Forest plot(A), leave-one-out sensitivity analysis(B) and funnel plot (C) of the effect of AS on TB-BMD(age 0-15); forest plot(D), leave-one-out sensitivity analysis(E) and funnel plot (F) of the effect of AS on TB-BMD(age 15-30); forest plot(G), leave-one-out sensitivity analysis(H) and funnel plot (I) of the effect of AS on TB-BMD(age 30-45); forest plot(J), leave-one-out sensitivity analysis(K) and funnel plot (L) of the effect of AS on TB-BMD(age 45-60); forest plot(M), leave-one-out sensitivity analysis(N) and funnel plot (O) of the effect of AS on TB-BMD(age over 60). AS: Ankylosing spondylitis; TB-BMD: total body bone mineral density.

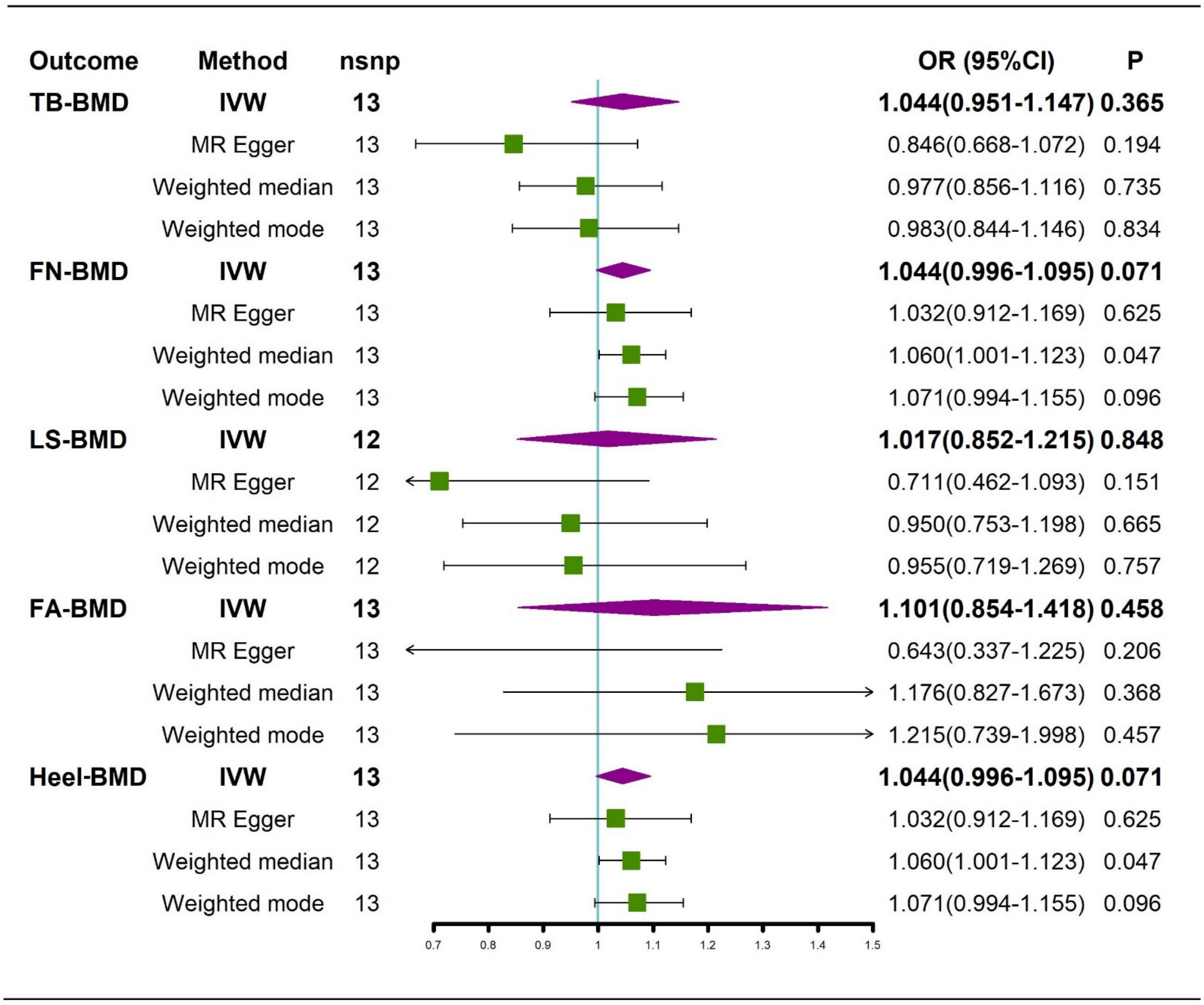

Supplementary Figure 3. MR analysis of AS on BMD by site using instrumental SNPs in or near genes that are functionally linked to AS.

AS: Ankylosing spondylitis; TB-BMD: total body bone mineral density; FN-BMD: femoral neck bone mineral density; LS-BMD: lumbar spine bone mineral density; FA-BMD: forearm bone mineral density; Heel-BMD: heel bone mineral density; IVW, inverse variance weighted; nsnp, number of single nucleotide polymorphisms; CI, confidence interval.

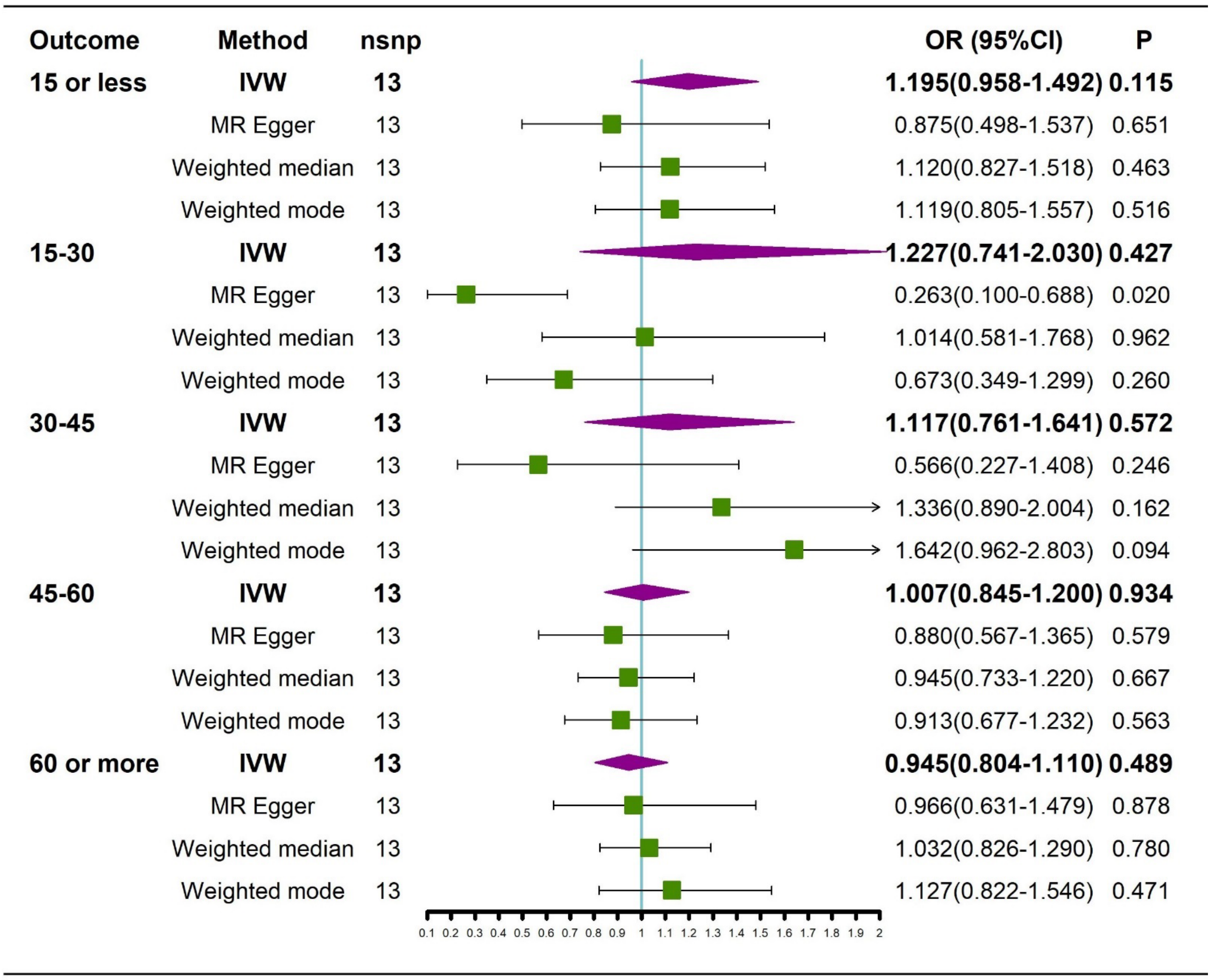

Supplementary Figure 4. MR analysis of AS on BMD at different ages using instrumental SNPs in or near genes that are functionally linked to AS.

AS: Ankylosing spondylitis; SNP, single nucleotide polymorphism; IVW, inverse variance weighted; nsnp, number of single nucleotide polymorphisms; CI, confidence interval.

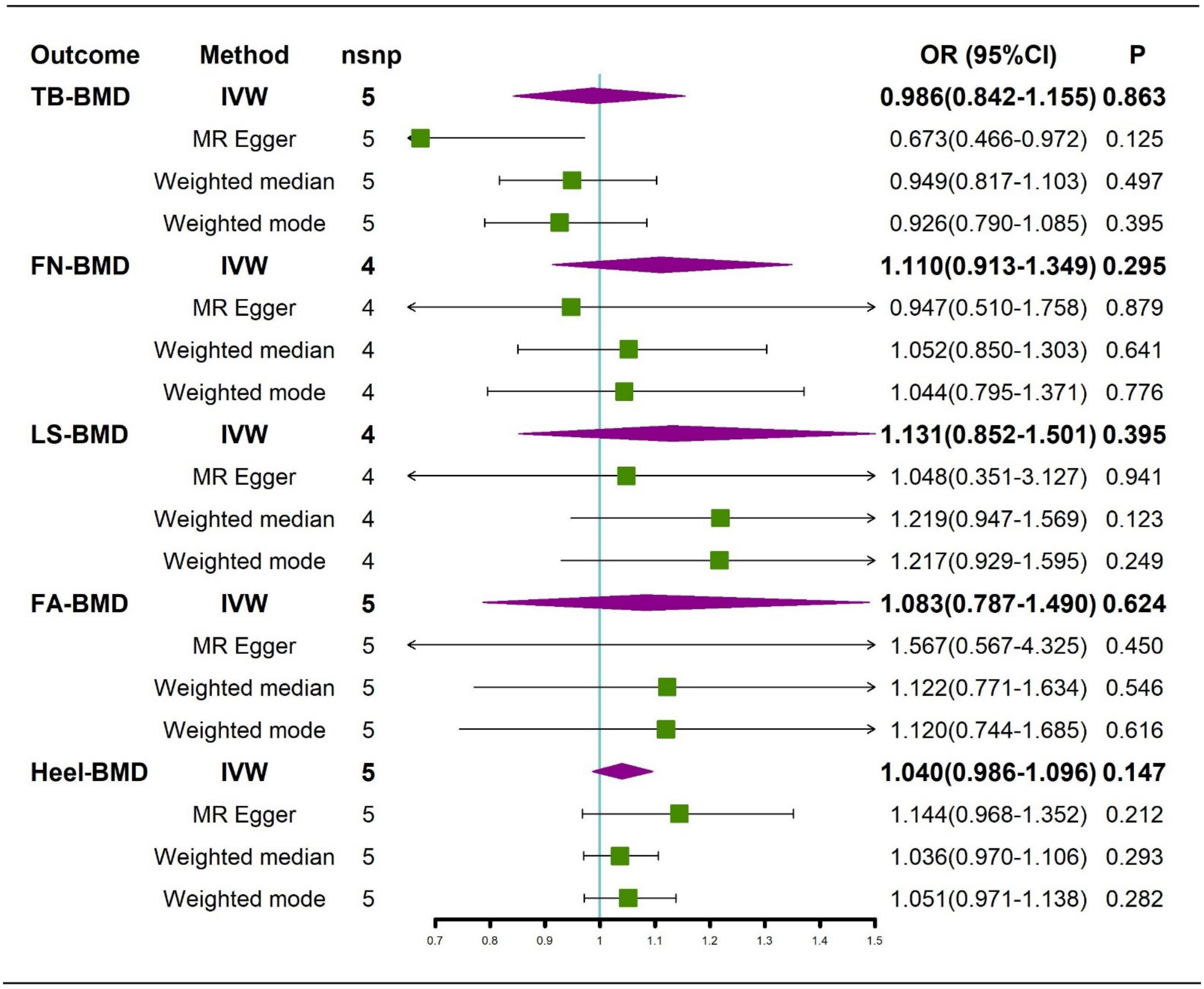

Supplementary Figure 5. Causal effects of AS on BMD at different sites after removal of potentially pleiotropic SNPs.

AS: Ankylosing spondylitis; FN-BMD: femoral neck bone mineral density; LS-BMD: lumbar spine bone mineral density; TB-BMD: total body bone mineral density; FA-BMD: forearm bone mineral density; Heel-BMD: heel bone mineral density; IVW, inverse variance weighted; nsnp, number of single nucleotide polymorphisms; CI, confidence interval.

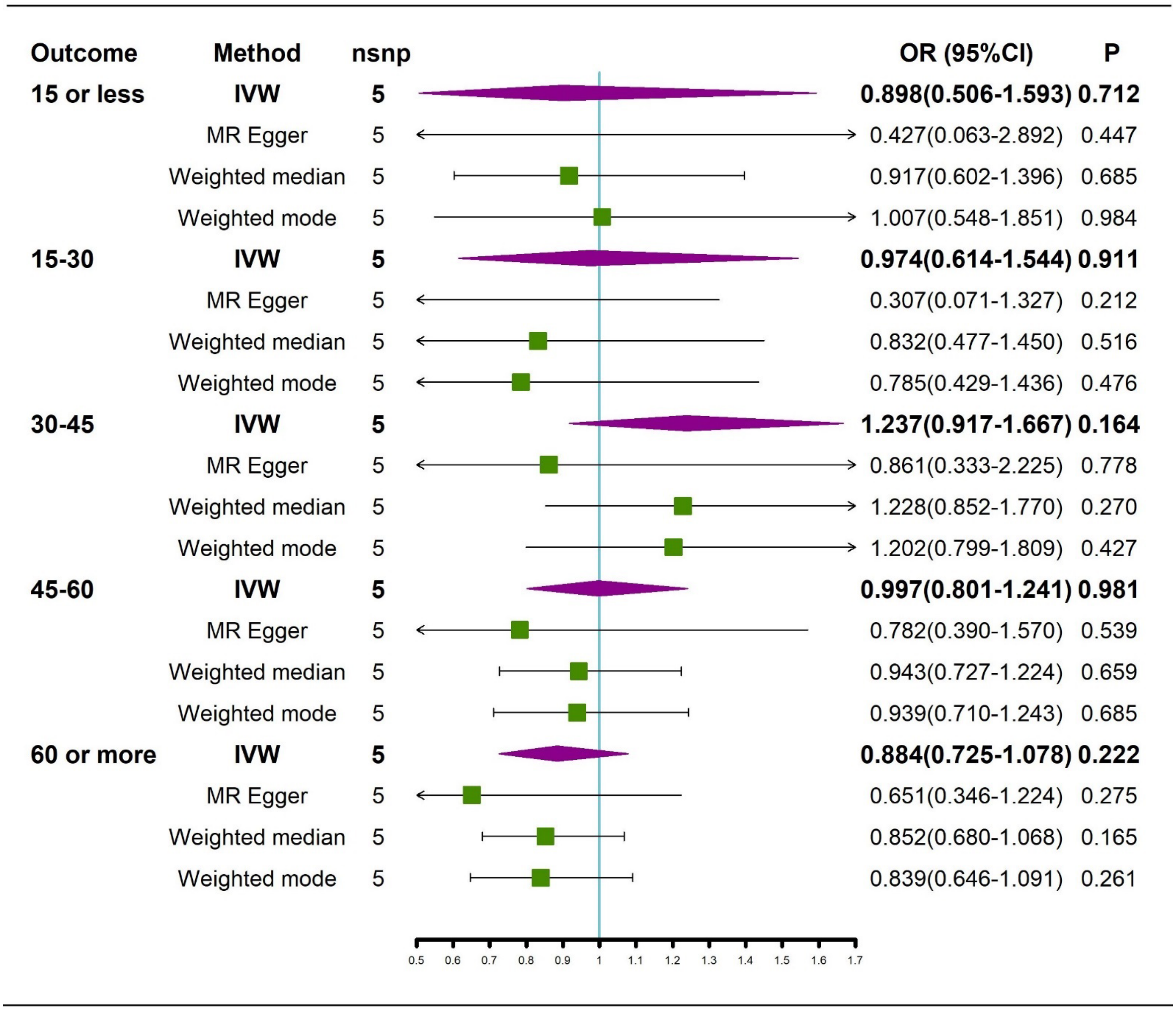

Supplementary Figure 6. Causal effects of AS on BMD in different age groups after removal of potentially pleiotropic SNPs.

AS: Ankylosing spondylitis; BMD: bone mineral density; IVW, inverse variance weighted; nsnp, number of single nucleotide polymorphisms; CI, confidence interval.

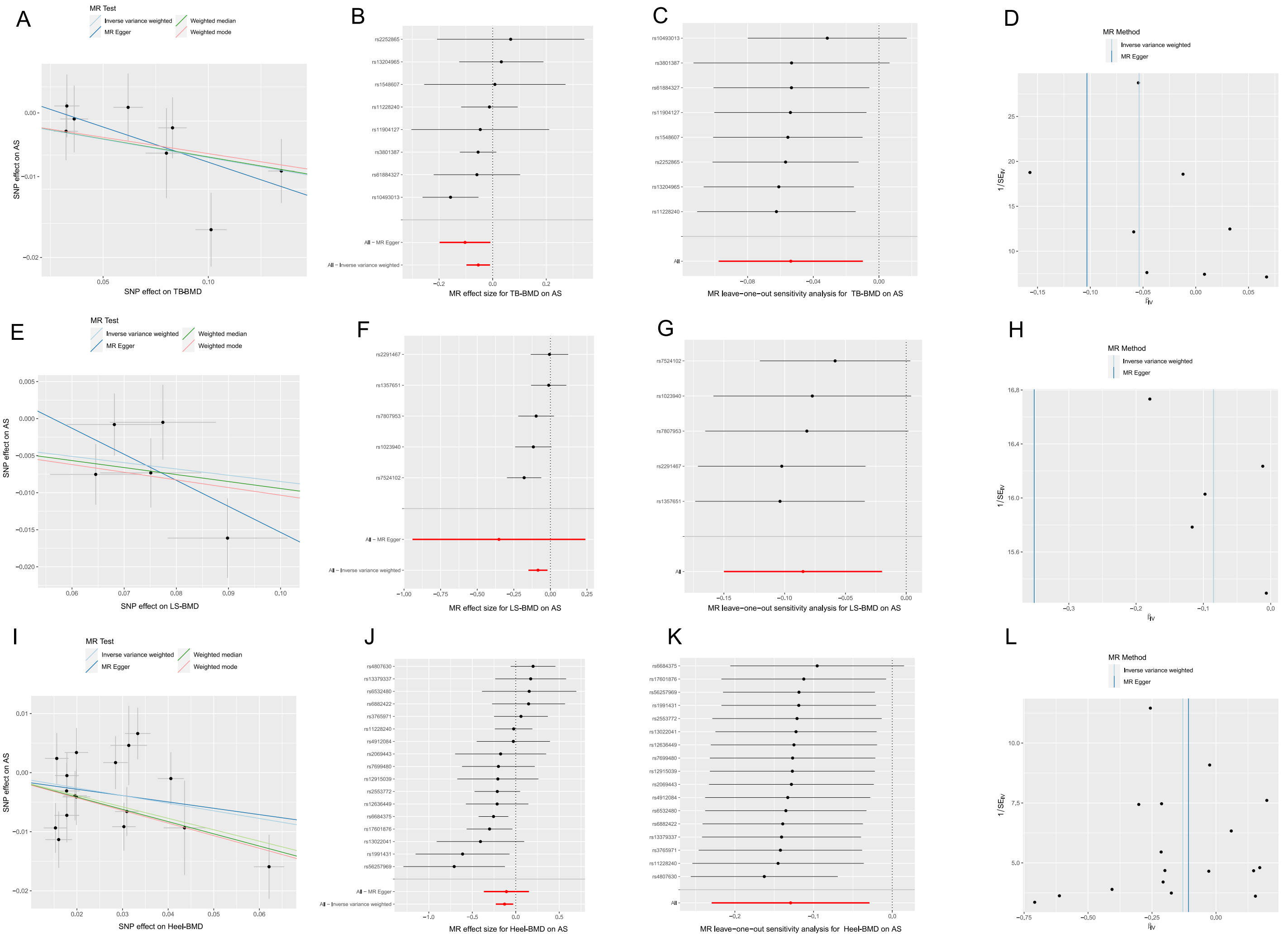

Supplementary Figure 7. MR sensitivity analysis of BMD on AS

Scatter plot(A), forest plot(B),leave-one-out sensitivity analysis (C) and funnel plot (D) of the effect of TB-BMD on AS; scatter plot(E), forest plot(F),leave-one-out sensitivity analysis (G) and funnel plot (H) of the effect of LS-BMD on AS; scatter plot(I), forest plot(J),leave-one-out sensitivity analysis (K) and funnel plot (L) of the effect of Heel-BMD on AS. AS: Ankylosing spondylitis; TB-BMD: total body bone mineral density; LS-BMD: lumbar spine bone mineral density; Heel-BMD: heel bone mineral density.

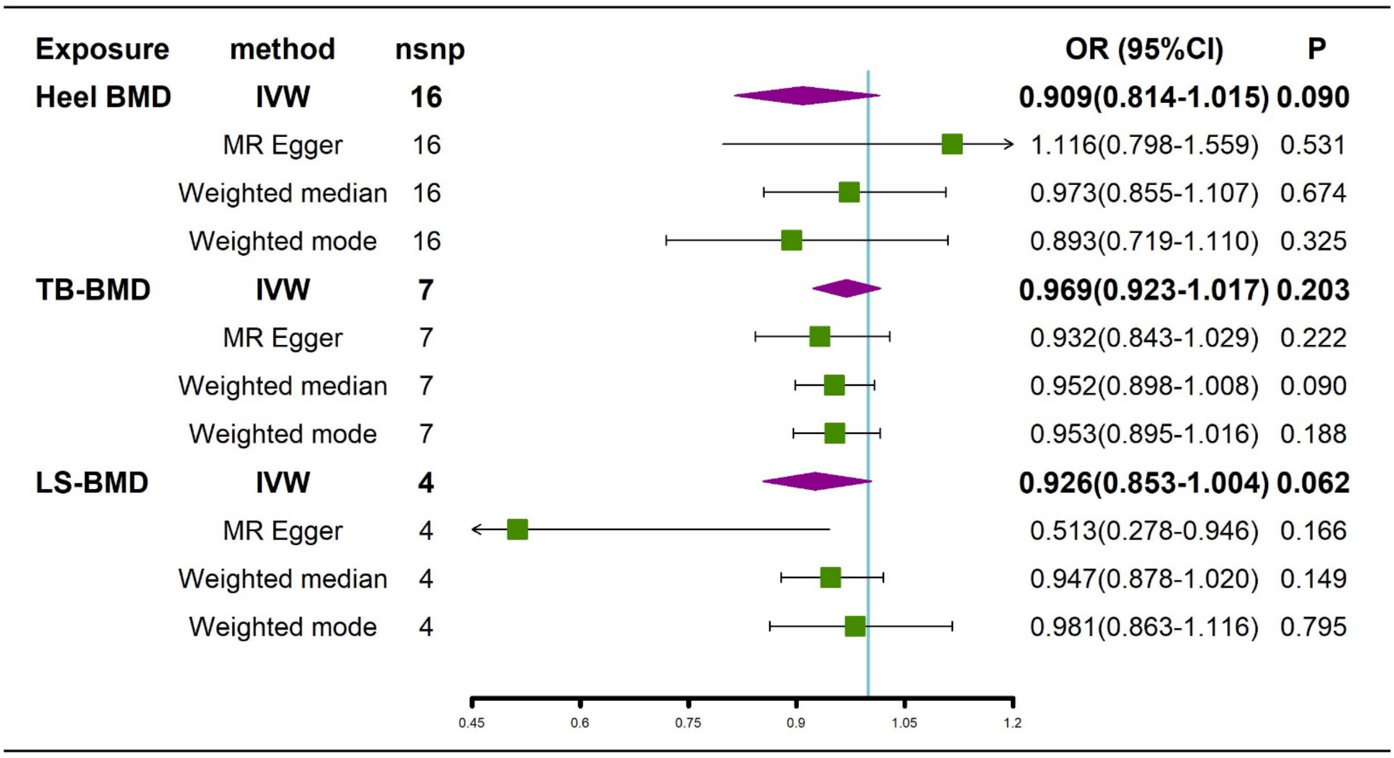

Supplementary Figure 8. Mendelian randomization analysis results for the effects of BMD on AS after removal of potentially pleiotropic SNPs.

AS: Ankylosing spondylitis; LS-BMD: lumbar spine bone mineral density; TB-BMD: total body bone mineral density; Heel-BMD: heel bone mineral density; IVW, inverse variance weighted; nsnp, number of single nucleotide polymorphisms; CI, confidence interval.
